# Silver(I) Complexes of Two Flexible Bis‐phospholane Ligands: Metallamacrocycles, Polymeric Chains, and Metallacryptands

**DOI:** 10.1002/zaac.202000001

**Published:** 2020-05-12

**Authors:** Paul Boar, Peter Lönnecke, Evamarie Hey‐Hawkins

**Affiliations:** ^1^ Faculty of Chemistry and Mineralogy Institute of Inorganic Chemistry Johannisallee 29 04103 Leipzig Germany

**Keywords:** Bis‐phospholane, Silver, Metallamacrocycles, Metallacryptand, Structure elucidation, Oligomers

## Abstract

In a 2:2 reaction with silver(I) chloride or bromide, 1,5‐bis(1‐phospholano)pentane (**1a**) afforded frame‐like macrocyclic structures, with intra‐ (**2**, Cl) or intermolecular (**3**, Br) halido bridges. In contrast, 1,7‐bis(1‐phospholano)heptane (**1b**) formed coordination polymers **4a** (Cl) and **4b** (Br) with bridging bis‐phospholane and halido ligands. A unique paddle wheel‐type metallacryptand structure **5** was obtained from **1a** and silver(I) bromide in a 2:3 reaction (M:L). All complexes were fully characterized by NMR, IR spectroscopy, mass spectrometry, and X‐ray crystallography.

## Graphical abstract

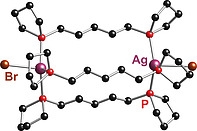

## Introduction

Phospholanes are five‐membered cyclic phosphines with good σ‐donor properties, which make them especially interesting as ligands in transition metal catalysis. Their ring structure and well‐defined geometry are key features, which set them apart from other phosphines.^1^ The first phospholane derivative was reported in 1916 by *Grüttner* and *Krause*. They synthesized 1‐phenylphospholane by reacting the di‐Grignard reagent of 1,4‐dibromobutane with dichlorophenylphosphine.^2^ More than 70 years passed before *Brunner* et al. published the synthesis of the chiral 3,4‐disubstituted correspondent.^3^ In 1990, both *Wilson* et al.^4^ and *Burk* et al.^5^ achieved the synthesis of enantiopure 2,5‐dimethyl‐substituted phospholanes starting from enantiopure 2,5‐hexanediol. The obtained chiral phospholanes proved very successful in asymmetric catalysis^6^ due to the close proximity of the chiral information to the catalytically active metal center. An interesting alternative for the synthesis of phospholanes starting from benzothiadiphosphole was reported by *Baccolini* et al.^7^ Both 1*H*‐phospholane and P‐substituted phospholanes can be obtained using this method. In 2009, the same group reported the successful synthesis of chiral alkylphospholanes.^8^

The real success story of the phospholane ligands started, however, in 1990 with the synthesis of bis‐phospholanoethane (BPE) and DuPhos by *Burk* and co‐workers.^4^, ^5^, ^9^ These simple bis‐phospholanes proved extremely efficient and versatile in rhodium‐catalysed asymmetric hydrogenation reactions, providing high activity and selectivity for a wide range of substrates.^10^ Following the success of these milestone ligands, the number of published bis‐phospholane ligands skyrocketed in the following years. In 2000, *Burk* published a paper entitled “Modular Phospholane Ligands in Asymmetric Catalysis”, which shows the ten‐year development of ligands based on the *trans*‐2,5‐disubstituted phospholane moiety.^6^ Four years later, *Clark* and *Landis* comprehensively summarized the following developments in this field.^11^ Together, these two reviews present the diversity of chiral phospholane and bis‐phospholane ligands and their application in a wide array of catalytic reactions.

These chiral bis‐phospholanes are usually based on rigid spacers that allow them to act as chelate ligands forming *cis* complexes with catalytically active metals.^6^, ^11^ Therefore, the number of atoms between the two phospholane moieties is, in general, no greater than three. Before 2014, examples of bis‐phospholane ligands with a longer and more flexible backbone were rare. 1,4‐Bis(1‐phospholano)butane was reported by *Emrich* and *Jolly*,^12^ and its chiral correspondent, 1,4‐bis(2,5‐diphenylphospholano)butane, was obtained by *Oisaki* et al.^13^ In both cases, however, the coordination chemistry of these ligands was not explored. Eventually, we reported a series of bis‐phospholane ligands with flexible alkylene spacers incorporating up to eleven methylene groups and showed that the number of carbon atoms in the chain directly influences their coordination behavior towards [AuCl(tht)] (tht = tetrahydrothiophene). When the chain consists of an odd number of carbon atoms, the formation of macrocyclic complexes (nanoframes) was observed, while for an even number of carbon atoms either polymeric chains or nanotubes were obtained.^14^ Two years later, in 2016, we reported the facile synthesis of silver(I) bis‐phospholane macrocycles using AgBF_4_ as a metal complex precursor.^15^ A comparison was made between one of the silver(I) macrocycles and the previously reported isomorphous gold(I) complex^14^ which gave further evidence that gold(I) is significantly smaller than silver(I). In 2019, a tridentate phosphine ligand with two phospholane end groups and a central phenylphosphino group connected by propylene spacers was shown to stabilize a molybdenum(0) complex in synthetic nitrogen fixation.^16^ Furthermore, in 2017 we reported the selective synthesis of two types of copper(I) metallamacrocycles using 1,5‐bis(1‐phospholano)pentane and [Cu(CH_3_CN)_4_]BF_4_.^17^

We have performed an in‐depth study of the coordination behavior of these flexible bis‐phospholane ligands in silver(I) halide complexes and report here the synthesis, characterization, and structural elucidation of complexes based on 1,5‐bis(1‐phospholano)pentane (**1a**)^14^ or 1,7‐bis(1‐phospholano)heptane (**1b**)^14^ and AgX (X = Cl, Br).

## Results and Discussion

**Scheme 1:**
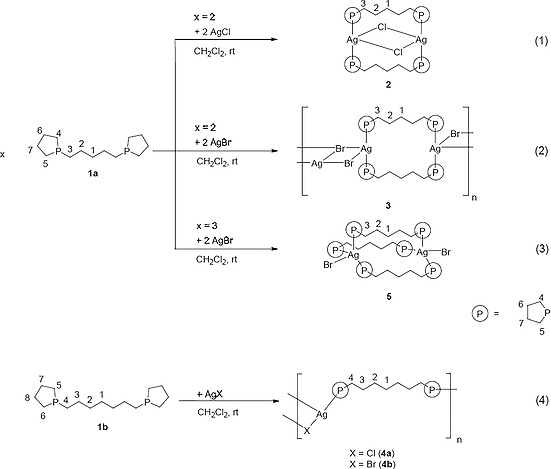
Synthesis of silver(I) bis‐phospholane complexes.

Equimolar reactions between 1,5‐bis(1‐phospholano)pentane (**1a**)^14^ and silver(I) chloride and bromide were performed in dichloromethane (DCM). The reaction mixtures were shielded from light and stirred at room temperature for four hours. In both cases, the ^31^P{^1^H} NMR spectrum of the crude reaction mixture showed the expected downfield shift from the free ligands (ca. –27 ppm)^14^ and only one sharp signal at approximately –7 ppm, indicative of a selective synthesis. Crystals of **2** and **3** suitable for X‐ray structure analysis were obtained at room temperature by slow diffusion of *n*‐hexane into a DCM/toluene solution. The results showed two different coordination modes: A metallacycle **2** with intramolecular chlorido bridges [Scheme 1 (1)], and metallacycles connected by intermolecular bromido bridges **3** [Scheme 1 (2)].

**Figure 1:**
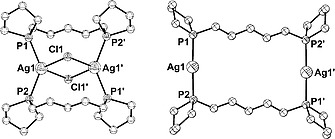
Molecular structure of complex **2** (left) and the corresponding complex based on AgBF_4_ (right).^15^ Hydrogen atoms and [BF_4_]^–^ counterions (right) are omitted for clarity.

Complex **2** crystallizes in the triclinic space group *P* 1 with one centrosymmetric molecule in the unit cell. Two silver(I) cations are bridged by two bis‐phospholane ligands forming a 16‐membered macrocycle, as was also observed in the reaction of **1a** with AgBF_4_ (Figure 1).^15^ However, the differences between the two structures are immediately apparent. When the weakly coordinating [BF_4_]^–^ anion is used, an almost linear coordination at silver(I) with a P–Ag–P bond angle of 176.89(1)° is observed resulting in an almost rectangular macrocycle,^15^ while the Cl^–^ anions form intramolecular chlorido bridges, which force the silver atoms closer together, resulting in an hourglass‐like shape of complex **2**. Consequently, the P–Ag–P bond angle is significantly smaller [137.76(7)°] (Table 1). The distortion is also noticeable when looking at the Ag–P bond lengths in **2** [245.5(2) and 245.7(2) pm] which are more than 8 pm longer than those in the corresponding AgBF_4_ complex.^15^ The chlorido‐bridged Ag_2_Cl_2_ moiety forms an almost perfect square as shown by Ag–Cl bond lengths of 271.1(2) and 270.9(2) pm and a Cl1–Ag1–Cl1' bond angle of 90.00(5)°. A similar structure was reported for the 2:2 complexes of 1,5‐bis(diphenylphosphanyl)pentane (dpppn) with AgX (X = Br, I) by *Effendy* et al.^18^ and with AgCl by *Cassel*.^19^ The Ag–P bond lengths in the dpppn complexes are about 2–4 pm longer than in complex **2**, which is to be expected given the electron‐withdrawing effect of the phenyl groups of the triphenylphosphine.

**Table 1:** Selected bond lengths /pm and angles /° in complex **2**

Bond lengths		Bond angles	
Ag1–P1	245.5(2)	P1–Ag1–P2	137.76(7)
Ag1–P2	245.7(2)	P1–Ag1–Cl1	96.07(6)
Ag1–Cl1	271.1(2)	P1–Ag1–Cl1'	113.37(6)
Ag1–Cl1'	270.9(2)	P2–Ag1–Cl1	113.98(6)
		P2–Ag1–Cl1'	96.33(6)
		Cl1–Ag1–Cl1'	90.00(5)

The ^31^P{^1^H} NMR spectrum of complex **2** in CDCl_3_ shows a singlet at *δ* = –5.7 ppm. Coupling with the NMR‐active silver isotopes ^107^Ag (*NA* = 51.84 %) or ^109^Ag (*NA* = 48.16 %; both I = ½) is not observed. The lack of coupling in solution at room temperature was also observed by *Streitberger* et al.^15^ and earlier by *Effendy* and co‐workers who carried out more thorough NMR studies on silver(I) bis(diphenyl)phosphine complexes.^18^ The stability of macrocycle **2** in solution is evidenced by the ESI(+) mass spectrum which showed the [M–Cl]^+^ molecular ion at *m/z* = 739.1.

**Figure 2:**
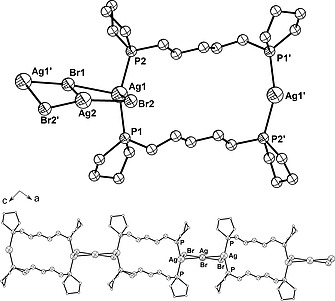
(Top) Section of the polymeric structure of coordination polymer **3** showing the homobimetallic macrocycle and the Ag_3_Br_3_ assembly. (Bottom) Polymeric chain along (101) in the crystal structure of **3**; phospholane carbon atoms are shown as white circles. Hydrogen atoms are omitted for clarity.

Complex **3** crystallizes in the monoclinic space group *C*2/*c* with four molecules in the unit cell. The solid‐state structure shows two molecules of 1,5‐bis(1‐phospholano)pentane bridging two silver(I) cations forming a frame‐like macrocycle [Ag–P 239.72(7) and 240.50(7) pm, P–Ag–P 153.94(2)°] (Table 2 and Figure 2). *Streitberger* et al. reported comparable Ag–P bond lengths of 237.21(8) and 237.02(8) pm but a significantly larger P–Ag–P bond angle of 176.89(1)° for a distinct frame‐like structure obtained using AgBF_4_ (Figure 1, right).^15^ A highly distorted tetrahedral environment around the metal center is created by two phospholane moieties and two bromine atoms. In contrast to the intramolecular chlorido bridges observed in **2**, here, the silver(I) cations of neighboring metallamacrocycles interact with an additional AgBr moiety, thus forming Ag_3_Br_3_ assemblies (Figure 2).

**Table 2:** Selected bond lengths /pm and angles /° in coordination polymer **3**

Bond lengths		Bond angles	
Ag1–P1	239.72(7)	P1–Ag1–P2	153.94(2)
Ag1–P2	240.50(7)	P1–Ag1–Br1	109.28(2)
Ag1–Br1	284.05(2)	P1–Ag1–Br2	92.08(2)
Ag1–Br2	301.22(4)	P2–Ag1–Br1	94.49(2)
Ag2–Br1	272.10(6)	P2–Ag1–Br2	96.87(2)
Ag2–Br2'	254.67(4)	Br1–Ag1–Br2	94.00(1)
		Br1–Ag2–Br2	108.71(1)
		Ag1–Br1–Ag1'	155.40(2)

Ag*_x_*Br*_y_* assemblies or clusters are known in silver(I) phosphine complexes. Often they form tetrameric cubane‐like structures (Ag_4_Br_4_), when simple monodentate phosphine ligands are employed.^20^ *Effendy* et al. reported a tetranuclear 2:1 complex (AgX:dppm; X = Cl, Br), which is formed when AgX and bis(diphenylphosphanyl)methane (dppm) are reacted in a 2:1 ratio. The structure of the complex includes an Ag_4_Br_4_ assembly with a “step” conformation.^21^ *Schubert* et al. found that dppm and AgBr can also form a 3:3 complex bearing an [Ag_3_Br_2_]^+^ cluster, while bis(diphenylphosphanyl)methylamine (dppa) forms a dimeric 2:4 complex in which an [Ag_4_Br_2_]^2+^ octahedron is present.^22^ A similar trimeric Ag_3_Br_3_ assembly to the one found in complex **3** was assumed to bridge adjacent macrocycles in a silver(I) complex based on 2,5‐bis(diphenylphosphinomethyl)thiophene (dpmt). However, only the crystal structure of the iodine homologue was reported.^23^ The solid‐state structure of complex **3** shows that Ag_3_Br_3_ bicycles are bridged by a set of two 1,5‐bis(1‐phospholano)pentane ligands, building a polymeric chain along (101) (Figure 2). Ag–Br distances range from 254.67(4) (Ag2–Br2) to 301.22(4) pm (Ag1–Br2) (Table 2) and are well inside the sum of the van der Waals radii (Σ_vdW_ Ag–Br = 390 pm).^24^ The dihedral angle between the planes described by the two squares, which form the bicycle Ag1–Br1–Ag2–Br2 and Ag2–Br1–Ag1'–Br2' (Figure 2, top), is 12.17(1)°. This torsion together with the distorted tetrahedral environment around the silver(I) cations force consecutive metallamacrocycles to adopt a twisted relative orientation. The dihedral angle between the mean planes described by neighboring metallamacrocycles has a value of 38.28(1)°.

The ^31^P{^1^H} NMR spectrum of complex **3** in CDCl_3_ shows a singlet at *δ* = –8.5 ppm. The ESI(+) mass spectrum showed peaks at *m/z* = 1590.6, 1402.8, 1158.7, and 1215.0, corresponding to the fragments [M+L+2Ag+Br]^+^, [M+L+Ag]^+^, [M+Ag]^+^, and [3 L+3Ag+2Br]^+^, respectively. The peaks at *m/z* = 970.9 (base peak) and 780.0 corresponding to fragments [M–Br]^+^ and [M–AgBr_2_]^+^, respectively, are proof that the frame‐like metallamacrocycle is stable in solution (M = L_2_Ag_3_Br_3_, L = **1a**).

Further complexation reactions between 1,7‐bis(1‐phospholano)heptane (**1b**)^14^ and silver(I) chloride and bromide were performed in DCM with a ligand:metal ratio of 1:1 using similar conditions as those previously described for ligand **1a**. The reaction mixtures were shielded from light. After stirring at room temperature for four hours, the ^31^P{^1^H} NMR spectra of the crude reaction mixture showed the expected downfield shift with signals around –7 ppm. Crystals of the final products **4a** and **4b** [Scheme 1 (4)] suitable for X‐ray structure analysis were obtained at room temperature by slow diffusion of *n*‐hexane into a DCM/THF solution (for **4a**) or a DCM solution (**4b**) of the crude product. The results showed similar coordination modes, namely the formation of coordination polymers with bridging bis‐phospholane and halido ligands.

**Figure 3:**
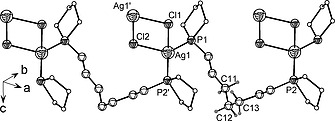
Chain of the coordination polymer **4a** showing geminally bridged Ag_2_Cl_2_ moieties by 1,7‐bis(1‐phospholano)heptane. Hydrogen atoms are omitted for clarity.

Coordination polymer **4a** crystallizes in the monoclinic space group *C*2/*c* and is based on the same structural motif as observed in complex **2**, an Ag_2_Cl_2_ moiety bridged by two phospholane ligands. However, in contrast to **2**, in the structure of **4a** the phosphorus atoms of one molecule of phospholane ligand are coordinating different Ag_2_Cl_2_ units forming polymeric chains (Figure 3). Cl1 and Cl2 are located on a twofold axis and all ligands are related by symmetry. The C_7_ alkylene spacer adopts a twisted V shape. The conformation of the C11–C12 and C12–C13 bonds is *gauche* while all other C–C bonds adopt a normal *anti* conformation, making the central atom C12 an inflection point (Figure 3). The flexibility of longer alkylene spacers has been discussed by *Effendy* et al. with regard to their adaptability to accommodate different combinations of ligand coordination modes. The authors found a greater variety of conformational possibilities in the structures of complexes based on 1,6‐bis(diphenylphosphanyl)hexane (dpph) than in those bearing shorter alkylene moieties.^18^

Ag–P bond lengths in **4a** [244.4(1) and 243.7(1) pm] are marginally shorter than in complex **2**. One of the Ag–Cl bonds (Ag1–Cl2) is ca. 6 pm shorter than the one in **2**, causing the Ag_2_Cl_2_ ring to slightly deviate from the ideal square arrangement, with angles ranging from 85.54(4) to 93.24(3)° (Table 3). The angles around silver(I) are ranging from approximately 93° to 133° and indicate a highly distorted tetrahedral environment.

**Figure 4:**
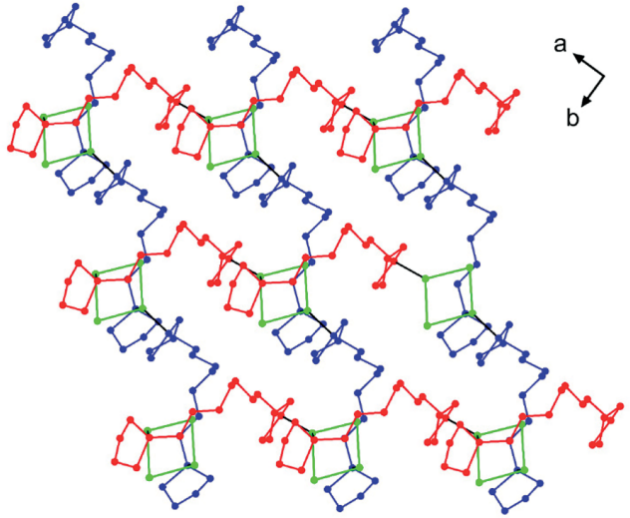
Interwoven chains [(110) chains in red; (110) chains in blue] of a (001) layer of structure **4a**. Ag–Cl groups are presented in green. Hydrogen atoms are omitted for clarity.

Coordination polymer **4a** forms a (001) layer structure in which the silver(I) cations are linked in series by chlorine atoms, while the bis‐phospholane ligands are arranged in a zigzag fashion. The layers are formed by two distinct interwoven polymeric chains (represented in red and blue in Figure 4), which zigzag across the layer and intersect in the nodes created by the Ag–Cl bridges (drawn in green).

The ^31^P{^1^H} NMR spectrum of the coordination polymer **4a** in CDCl_3_ shows a singlet at *δ* = –5.3 ppm. The ESI(+) mass spectrum showed the base peak at *m/z* = 795 corresponding to the fragment [L_2_Ag_2_Cl]^+^ (L = ligand **1b**).

**Figure 5:**
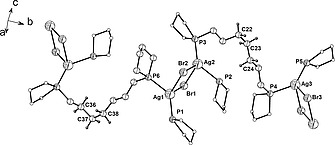
Chain of the coordination polymer **4b** showing Ag_2_Br_2_ moieties vicinally bridged by 1,7‐bis(1‐phospholano)heptane. Hydrogen atoms are omitted for clarity.

Complex **4b** crystallizes in the monoclinic space group *P*2_1_/*c*. The solid‐state structure (Figure 5) shows a coordination polymer similar to its AgCl‐based homologue **4a** (Figure 3). However, using the concept of interwoven chains, in this case 1,7‐bis(1‐phospholano)heptane acts as a bridging ligand and connects neighboring Ag_2_Br_2_ assemblies in a vicinal fashion forming a polymeric (102) layer (Figure 5).

Each metal center is coordinated by two phospholane moieties and two bromide atoms, which create a distorted tetrahedral arrangement. The angles around the silver(I) cations range from approximately 89° to 134° (Table 4) and are comparable to those discussed for coordination polymer **3**. The P–Ag–P bond angles [133.03(3) to 138.70(3)°, Table 4] are significantly smaller than the ones in macrocycle **3** (Table 2) but comparable to those in complex **2** and coordination polymer **4a** (Table 3). The same trend was reported by *Effendy* et al. for flexible bis(diphenylphosphanyl)alkane ligands when comparing open macrocycles [P–Ag–P 144.30(7)°] to species in which halido bridging was observed [P–Ag–P 131.24(9)°].^17^ The Ag–P bond lengths in **4b** [241.0(1) to 244.9(1) pm, Table 4] are slightly larger than those in **3** (Table 2). As previously discussed for coordination polymer **4a**, the C_7_ alkylene spacer also adopts a V‐shape in **4b**. The conformation of the C22–C23, C23–C24, and C36–C37, C37–C38 bonds is *gauche*, making the central atoms C23 and C37 inflection points (Figure 5).

**Table 3:** Selected bond lengths /pm and angles /° in coordination polymer **4a**

Bond lengths		Bond angles	
Ag1–P1	244.4(1)	P1–Ag1–P2	132.93(4)
Ag1–P2	243.7(1)	P1–Ag1–Cl1	100.28(3)
Ag1–Cl1	270.8(1)	P1–Ag1–Cl2	109.27(2)
Ag1–Cl2	264.8(1)	P2–Ag1–Cl1	110.48(3)
		P2–Ag1–Cl2	101.61(3)
		Cl1–Ag1–Cl2	93.24(3)

**Table 4:** Selected bond lengths, distances /pm and angles /° in coordination polymer **4b**

Bond lengths		Bond angles	
Ag1–P1	241.0(1)	P1–Ag1–P6	133.03(3)
Ag1–P6	244.7(1)	P1–Ag1–Br1	121.74(3)
Ag2–P2	244.9(1)	P1–Ag1–Br2	109.13(3)
Ag2–P3	242.17(9)	P6–Ag1–Br1	94.54(3)
Ag3–P4	241.16(9)	P6–Ag1–Br2	89.83(3)
Ag3–P5	243.94(9)	Br1–Ag1–Br2	100.75(1)
Ag1–Br1	275.51(5)	P2–Ag2–P3	133.50(3)
Ag1–Br2	293.21(5)	P2–Ag2–Br2	96.08(3)
Ag2–Br2	276.93(5)	P2–Ag2–Br1	89.24(3)
Ag2–Br1	292.49(5)	P3–Ag2–Br2	96.08(3)
Ag3–Br3	284.52(4)	P3–Ag2–Br1	106.84(3)
Ag3–Br3'	287.24(7)	Br2–Ag2–Br1	100.59(1)
Ag1**···**Ag2	363.44(6)	P4–Ag3–P5	138.70(3)
Ag3**···**Ag3'	385.68(6)	P4–Ag3–Br3	89.57(2)
		P5–Ag3–Br3	122.29(3)

**Figure 6:**
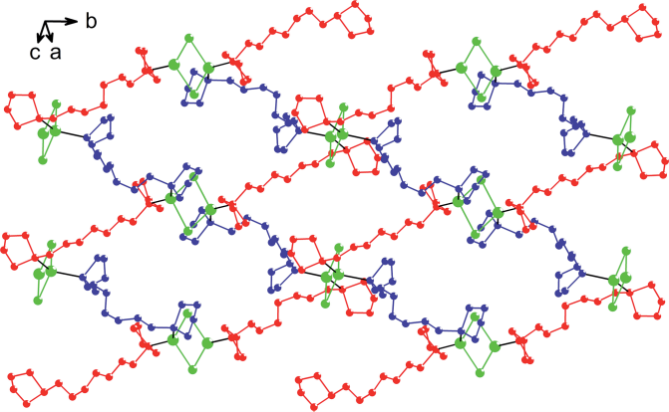
Interwoven chains (red and blue) of a (102) layer of structure **4b**. Ag–Br groups are presented in green. Hydrogen atoms are omitted for clarity.

There are two types of bromido bridges present in **4b** (Figure 6) The Ag–Br distances in the rhombic Ag1–Br1–Ag2–Br2 ring are 275.51(5)/293.21(5) (Ag1) and 276.93(5)/292.49(5)pm (Ag2), the ones in the Ag3–Br3–Ag3'–Br' ring are more similar with 284.52(4) and 287.24(7) pm. This influences the Ag**···**Ag distances, which have considerably different values of 363.44(6) and 385.68(6) ppm (Table 4).

The ^31^P{^1^H} NMR spectrum of coordination polymer **4b** in CDCl_3_ shows a singlet at *δ* = –5.7 ppm. The base peak in the ESI(+) mass spectrum at *m/z* = 839.1 corresponds to the fragment [L_2_Ag_2_Br]^+^ (L = ligand **1b**). Two other peaks corresponding to species with higher *m/z* values were observed but could not be attributed and probably correspond to oligomeric fragments.

**Figure 7:**
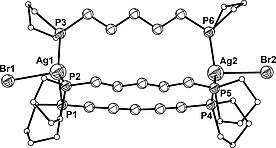
Molecular structure of complex **5**. Phospholane carbon atoms are shown as white circles and hydrogen atoms are omitted for clarity.

Reactions between three equivalents of bis‐phospholane **1a**,**1b** and two equivalents of AgBr were performed under similar conditions as described for the 1:1 reactions. The ^31^P{^1^H} NMR spectrum of the crude reaction mixtures showed, in each case, one downfield‐shifted broad singlet. Suitable crystals for X‐ray structure analysis were obtained at room temperature by layering a DCM/toluene (for ligand **1a**) or DCM (for ligand **1b**) solution of the crude product with *n*‐hexane. X‐ray structure analysis showed that in the case of ligand **1b** the same coordination polymer **4b** was formed as in the 1:1 reaction, while ligand **1a** yielded the metallacryptand **5** (Figure 7).

Complex **5** crystallizes in the orthorhombic space group *Pbca* with eight molecules in the unit cell and is the desired stoichiometric product. Two silver(I) cations are bridged by three 1,5‐bis(1‐phospholano)pentane ligands in a trigonal‐planar fashion forming a triangular prism (paddle wheel) with Ag–P distances ranging from 241.88(9) to 251.48(9) pm and P–Ag–P angles ranging from 104.74(3) to 137.51(3)° (Table 5). The lengths of four of the six Ag–P bonds are comparable to those observed for **4b**; one of the three Ag–P bonds in each moiety is longer, i.e. Ag1–P1 and Ag2–P4 [251.48(9) and 248.5(1) pm, respectively]. Ag–Br bond lengths [278.90(4) and 281.84(5) pm] are comparable to those previously observed in **4b**.

**Table 5:** Selected bond lengths /pm and angles /° in complex **5**

Bond lengths		Bond angles	
Ag1–P1	251.48(9)	P1–Ag1–P3	104.74(3)
Ag1–P3	244.51(9)	P1–Ag1–P2	113.96(3)
Ag1–P2	244.38(9)	P3–Ag1–P2	137.51(3)
Ag2–P4	248.5(1)	P1–Ag1–Br1	92.29(2)
Ag2–P6	243.95(9)	P3–Ag1–Br1	104.94(2)
Ag2–P5	241.88(9)	P2–Ag1–Br1	90.77(2)
Ag1–Br1	278.90(4)	P4–Ag2–P6	110.31(3)
Ag2–Br2	281.84(5)	P4–Ag2–P5	115.89(3)
		P6–Ag2–P5	131.70(3)
		P4–Ag2–Br2	89.80(2)
		P6–Ag2–Br2	94.24(2)
		P5–Ag2–Br2	99.13(2)

Silver(I) complexes with an M:L ratio of 2:3 and with bridging bis(phosphanyl)alkane ligands are rare. ^31^P{^1^H} NMR studies reported by *Dean* et al. (1987) and later by *Peringer* et al. (1988) postulated the formation of complexes [Ag_2_(μ‐dppm)_3_](AsF_6_)_2_ 25 and [Ag_2_(μ‐dppm)_3_](CF_3_SO_3_)_2_,^26^ respectively. Ten years later, *Hong* et al. published the structure of [Ag_2_(μ‐dppm)_3_](NO_3_)_2_ and revealed its paddle wheel topology.^27^ To the best of our knowledge, paddle wheel structures containing bis‐phosphine ligands with longer alkylene chains have not yet been reported. Therefore, complex **5** is not only the first such example bearing bis‐phospholane ligands but also the first silver(I) bis‐phosphine paddle wheel‐type metallacryptand.

## Conclusions

The reaction of silver(I) chloride or bromide with two highly flexible bis‐phospholane ligands, 1,5‐bis(1‐phospholano)pentane (**1a**) and 1,7‐bis(1‐phospholano)heptane (**1b**), gave macrocycles, coordination polymers and paddle wheel‐type metallacryptands depending on the stoichiometry, length of alkylene spacer (C_5_ or C_7_) and counter anion. These complexes are further proof that these simple and highly flexible bis‐phospholane ligands have extremely versatile coordination properties.

## Experimental Section

All reactions and crystallization of compounds were carried out in a nitrogen atmosphere by using standard Schlenk techniques^28^ and dry solvents. 1,5‐Bis(1‐phospholano)pentane^14^ and 1,7‐bis(1‐phospholano)heptane (**1b**)^14^ were prepared according to literature procedures. All other chemicals were used as purchased. NMR spectra were recorded at 298 K with a Bruker AVANCE DRX 400 spectrometer. The chemical shifts (*δ*) of ^1^H, ^13^C, and ^31^P are reported in parts per million (ppm) at 400.12, 100.63, and 162.02 MHz, respectively, with tetramethylsilane as internal standard and referencing to the unified Ξ scale.^29^ Coupling constants *J* are given in Hertz. The numbering scheme for assignment of ^1^H NMR spectra is given in Scheme 1. IR spectra were recorded with a Thermofisher Nicolet iS5 ATR spectrometer, scanning between 400 and 4000 cm^–1^. Wavenumbers ν̃ are reported in cm^–1^. Mass spectra were recorded with an ESQUIRE 3000 plus spectrometer. Elemental analyses were carried out with a Heraeus VARIO EL oven. Melting points were measured in sealed capillaries by using a variable heater from Gallenkamp.

**Synthesis of Bis[1,5‐bis(1‐phospholano)pentane)]disilver(I) dichloride (2):** AgCl (21.4 mg, 1 equiv.) was added as a solid to a stirred solution of **1a** (36.6 mg, 1 equiv.) in 10 mL DCM. The Schlenk flask was immediately wrapped in aluminum foil and the reaction mixture was stirred at room temperature for 4 h, filtered and all volatiles were removed in vacuo. Afterwards, the white residue was washed with *n*‐hexane (3 × 2 mL) to yield **2** (49 mg, 85 %) as a white solid. Crystals suitable for single‐crystal X‐ray diffraction were obtained from a DCM/toluene mixture layered with *n*‐hexane at room temperature. M.p. 129 °C. **^1^H NMR** (CDCl_3_): *δ* = 2.19–2.03 (m, 8 H, H3), 1.92–1.72 (m, 16 H, H4, H5), 1.72–1.59 (m, 8 H, H2), 1.50–1.48 ppm (br. s, 20 H, H1, H6, H7). **^13^C{^1^H} NMR** (CDCl_3_): *δ* = 32.3–32.0 (several s or m), 28.0–27.9 (several s or m), 27.2 (s), 27.1–26.9 (several s or m), 26.3–25.7 ppm (several s or m). **^31^P{^1^H} NMR** (CDCl_3_): *δ* = –5.7 ppm (s). **IR**: ν̃ = 2937 (m, νCH), 2915 (m, νCH), 2855 (m, νCH), 1455 (w, νPC), 1436 (w, νPC), 1113 (w), 843 (w), 689 cm^–1^ (w). **MS** (ESI(+), DCM/acetonitrile): *m/z* = 739.1 [M–Cl]^+^, 597.2 [AgL_2_]^+^, 351.1 [AgL]^+^; L = **1a**. C_26_H_52_Ag_2_Cl_2_P_4_: calcd. C 40.28, H 6.76 %; found C 40.29, H 6.82 %.

**Synthesis of Poly{bis[1,5‐bis(1‐phospholano)pentane]trisilver(I) tribromide} (3):** AgBr (28.2 mg, 1 equiv.) was added as a solid to a stirred solution of **1a** (36.6 mg, 1 equiv.) in 10 mL DCM. The Schlenk flask was immediately wrapped in aluminum foil and the reaction mixture was stirred at room temperature for 4 h, filtered and all volatiles were removed in vacuo. Afterwards the white residue was washed with *n*‐hexane (3 × 2 mL) to remove excess ligand, yielding **3** (50 mg, 95 % based on AgBr) as a white solid. Crystals suitable for single‐crystal X‐ray diffraction were obtained from a DCM solution layered with *n*‐hexane at room temperature. M.p. 195 °C. **^1^H NMR** (CDCl_3_): *δ* = 2.20–2.04 (m, 8 H, H3), 1.91–1.76 (m, 16 H, H4, H5), 1.75–1.55 ppm (m, 28 H, H1, H2, H6, H7). **^13^C{^1^H} NMR** (CDCl_3_): *δ* = 27.6–27.3 (several s or m), 27.1 (s), 26.3–26.0 (several s or m), 26.0–25.8 ppm (several s or m). **^31^P{^1^H} NMR** (CDCl_3_): *δ* = –8.5 ppm (s). **IR**: ν̃ = 2934 (m, νCH), 2919 (m, νCH), 2845 (m, νCH), 1444 (w, νPC), 1407 (w, νPC), 1112 (w), 1023 (w), 845 (w), 708 cm^–1^ (w). **MS** (ESI(+), DCM/MeOH): *m/z* = 1590.6 [M+L+2Ag+Br]^+^, 1402.8 [M+L+Ag]^+^, 1158.7 [M+Ag]^+^, 1215.0 [Ag_3_L_3_Br_2_]^+^, 970.9 [M–Br]^+^, 780.0 [M–AgBr_2_]^+^; M = [Ag_3_L_2_Br_3_], L = **1a**. C_26_H_52_Ag_3_Br_3_P_4_: calcd. C 29.69, H 4.98 %; found C 29.66, H 5.01 %.

**Synthesis of Poly{bis[1,7‐bis(1‐phospholano)heptane]silver(I) chloride} (4a):** AgCl (21.4 mg, 1 equiv.) was added as a solid to a stirred solution of **1b** (40.8 mg, 1 equiv.) in 10 mL DCM. The Schlenk flask was immediately wrapped in aluminum foil and the reaction mixture stirred at room temperature for 4 h, filtered and all volatiles were removed in vacuo. Afterwards the white residue was washed with *n*‐hexane (3 × 2 mL) to yield **4a** (51 mg, 82 %) as a white solid. Crystals suitable for single‐crystal X‐ray diffraction were obtained from a DCM/THF mixture layered with *n*‐hexane at room temperature. M.p. 103 °C. **^1^H NMR** (CDCl_3_): *δ* = 2.12–1.99 (m, 4 H, H4), 1.85–1.66 (m, 8 H, H5, H6), 1.64–1.53 (m, 4 H, H3), 1.51–1.14 (m, 14 H, H1, H2, H7, H8). **^13^C{^1^H} NMR** (CDCl_3_): *δ* = 31.1–30.5 (several s or m), 29.2 (s), 28.3–27.8 (several s or m), 27.2 (s), 27.1–26.8 (several s or m), 26.3–25.8 ppm (several s or m). **^31^P{^1^H} NMR** (CDCl_3_): *δ* = –5.3 ppm (s). **IR**: ν̃ = 2962 (m, νCH), 2927 (m, νCH), 2856 (m, νCH), 1446 (w, νPC), 1261 (s), 1105 (s), 1024 (s), 802 (s), 720 cm^–1^ (w). **MS** (ESI(+), DCM/MeOH): *m/z* = 795.0 [Ag_2_L_2_Cl]^+^, 651.0 [AgL_2_]^+^, 379.0 [AgL]^+^; L = **1b**. C_15_H_30_AgClP_2_: calcd. C 43.34, H 7.27 %; found C 43.30, H 7.21 %.

**Synthesis of Poly{bis[1,7‐bis(1‐phospholano)heptane]silver(I) bromide} (4b):** AgBr (28.2 mg, 1 equiv.) was added as a solid to a stirred solution of **1b** (40.8 mg, 1 equiv.) in 10 mL DCM. The Schlenk flask was immediately wrapped in aluminum foil and the reaction mixture was stirred at room temperature for 4 h, filtered and all volatiles were removed in vacuo. Afterwards the white residue was washed with *n*‐hexane (3 × 2 mL) to yield **4b** (68 mg, 98 %) as a white solid. Crystals suitable for single‐crystal X‐ray diffraction were obtained from a DCM solution layered with *n*‐hexane at room temperature. M.p. 150 °C. **^1^H NMR** (CDCl_3_): *δ* = 2.22–2.09 (m, 4 H, H4), 1.94–1.74 (m, 8 H, H5, H6), 1.70–1.60 (m, 4 H, H3), 1.54–1.22 ppm (m, 14 H, H1, H2, H7, H8). **^13^C{^1^H} NMR** (CDCl_3_): *δ* = 31.0–30.2 (several s or m), 29.1 (s), 28.4–27.7 (several s or m), 27.3 (s), 27.1–26.7 (several s or m), 26.5–25.8 ppm (several s or m). **^31^P{^1^H} NMR** (CDCl_3_): *δ* = –5.7 ppm (s). **IR**: ν̃ = 2931 (m, νCH), 2852 (m, νCH), 1447 (w, νPC), 1400 (w, νPC), 1113 (w), 846 cm^–1^ (w). **MS** (ESI(+), chloroform/MeOH): *m/z* = 839.1 [Ag_2_L_2_Br]^+^, 381.1 [AgL]^+^; L = **1b**. C_15_H_30_AgBrP_2_: calcd. C 39.16, H 6.57 %; found C 39.23, H 6.65 %.

**Synthesis of Tris[1,5‐bis(1‐phospholano)pentane]disilver(I) Dibromide (5):** AgBr (18.8 mg, 2 equiv.) was added as a solid to a stirred solution of **1a** (36.6 mg, 3 equiv.) in 10 mL DCM. The Schlenk flask was immediately wrapped in aluminum foil and the reaction mixture stirred at room temperature for 4 h, filtered and all volatiles were removed in vacuo. Afterwards the white residue was washed with *n*‐hexane (3 × 2 mL) to yield **5** (53 mg, 98 %) as a white solid. Crystals suitable for single‐crystal X‐ray diffraction were obtained from a DCM/toluene mixture layered with *n*‐hexane at room temperature. M.p. 139 °C. **^1^H NMR** (CDCl_3_): *δ* = 2.18–2.05 (m, 12 H, H3), 1.94–1.81 (m, 12 H, H4), 1.79–1.68 (m, 12 H, H5), 1.60–1.48 (m, 12 H, H2), 1.45–1.38 ppm (m, 30 H, H1, H6, H7). **^13^C{^1^H} NMR** (CDCl_3_): *δ* = 34.0–33.4 (several s or m), 29.3 (s), 27.9 (d, ^1^ *J*_C,P_ = 10.7 Hz), 27.4 (s), 27.0 ppm (d, ^2^ *J*_C,P_ = 7.6 Hz). **^31^P{^1^H} NMR** (CDCl_3_): *δ* = –9.1 ppm (s). **IR**: ν̃ = 2921 (m, νCH), 2855 (m, νCH), 1447 (w, νPC), 1409 (w, νPC), 1109 (w), 1069 (w), 848 cm^–1^ (w). **MS** (ESI(+), DCM/MeOH): *m/z* = 783.0 [Ag_2_L_2_Br]^+^, 595.2 [AgL_2_]^+^; L = **1a**. C_39_H_78_Ag_2_Br_2_P_6_: calcd. C 42.26, H 7.09 %; found C 42.28, H 7.04 %.

**Crystal Structure Determinations:** The data were collected on a Gemini diffractometer (Rigaku Oxford Diffraction) using Mo‐*K*_α_ radiation (*λ* = 71.073 pm) and ω‐scan rotation. Data reduction was performed with CrysAlisPro^30^ including the program SCALE3 ABSPACK for empirical absorption correction. The structures were solved by direct methods (**2**: SHELXS‐97;^31^ **4a**: SIR‐92^32^) and dual space methods (**3**, **4b**, **5**: SHELXT‐2014^33^). The refinement of all non‐hydrogen atoms was performed with SHELXL‐2018.^34^ Except disordered parts of a structure, all non‐hydrogen atoms were refined with anisotropic thermal parameters. Hydrogen atoms were calculated on idealized positions using the riding model. Structure figures were generated with DIAMOND‐4.^35^ With the exception of **4a**, all structures are disordered to a certain extent. Mostly the five‐membered C_4_H_8_P ring was disordered, and for **3** and **4b** some of the C_n_H_2n_ chains were disordered as well. Details concerning data collection and crystallographic details are summarized in Table 6.

**Table 6:** Data collection and crystallographic data for compounds **2**, **3**, **4a**, **4b**, and **5**

	**2 ^a^** ^)^	**3**	**4a**	**4b**	**5**
Molecular formula	C_26_H_52_Ag_2_Cl_2_P_4_	C_26_H_52_Ag_3_Br_3_P_4_	C_15_H_30_AgClP_2_	C_15_H_30_AgBrP_2_ **·**CH_2_Cl_2_	C_39_H_78_Ag_2_Br_2_P_6_ **·**CH_2_Cl_2_
Empirical formula	C_26_H_52_Ag_2_Cl_2_P_4_	C_26_H_52_Ag_3_Br_3_P_4_	C_15_H_30_AgClP_2_	C_16_H_32_AgBrCl_2_P_2_	C_40_H_80_Ag_2_Br_2_Cl_2_P_6_
Formula weight	775.19	1051.89	415.65	545.03	1193.32
Temperature [K]	130(2)	130(2)	130(2)	130(2)	130(2)
Wavelength	71.073 pm	71.073 pm	71.073 pm	71.073 pm	71.073 pm
Crystal system	triclinic	monoclinic	monoclinic	monoclinic	orthorhombic
Space group	*P* 1	*C*2/*c*	*C*2/*c*	*P*2_1_/*c*	*Pbca*
Unit cell dimensions					
*a* /pm	868.88(9)	2057.39(7)	1455.94(4)	1315.45(1)	1633.10(2)
*b* /pm	882.5(1)	1098.73(3)	1034.29(3)	2229.57(2)	2167.83(3)
*c* /pm	1058.99(9)	1587.35(6)	2400.28(7)	2285.83(3)	2938.30(4)
*α* /°	73.86(1)	–	–	–	–
*β* /°	82.108(8)	98.122(3)	91.952(3)	101.432(1)	–
*γ* /°	86.02(1)	–	–	–	–
Volume /nm^3^	0.7722(2)	3.5522(2)	3.6124(2)	6.5711(1)	10.4024(2)
Z	1	4	8	12	8
*ρ*_calcd_ /Mg**·**m^–3^	1.667	1.967	1.529	1.653	1.524
*μ* /mm^–1^	1.664	5.208	1.428	3.130	2.603
*F*(000)	396	2056	1712	3288	4864
Crystal size /mm^3^	0.1 × 0.05 × 0.03	0.20 × 0.15 × 0.02	0.10 × 0.10 × 0.04	0.30 × 0.20 × 0.06	0.35 × 0.30 × 0.03
*Θ*_Min_ – *Θ*_Max_ /°	2.916–25.026	2.000–32.670	1.698–26.369	1.818–32.270	1.864–28.373
Index ranges	–10 ≤ *h* ≤ 10	–30 ≤ *h* ≤ 30	–18 ≤ *h* ≤ 18	–19 ≤ *h* ≤ 19	–21 ≤ *h* ≤ 21
	–10 ≤ *k* ≤ 10	–16 ≤ *k* ≤ 15	–12 ≤ *k* ≤ 12	–33 ≤ *k* ≤ 33	–28 ≤ *k* ≤ 28
	–12 ≤ *l* ≤ 12	–23 ≤ *l* ≤ 23	–29 ≤ *l* ≤ 29	–31 ≤ *l* ≤ 33	–38 ≤ *l* ≤ 37
Reflections collected	8374	25108	14198	103212	97671
Independent reflections {R_int_}	8374 {twin}	5971 {0.0398}	3691 {0.0558}	22073 {0.0473}	12308 {0.0711}
Completeness {*Θ*_Max_}	99.8 % {25.03}	100.0 % {30.51}	100.0 % {26.37}	100.0 % {30.51}	100.0 % {28.37}
T_Max_ / T_Min_	1.0000 / 0.9103	1.0000 / 0.5241	1.0000 / 0.9679	1.0000 / 0.8060	1.0000 / 0.7420
Restraints / parameters	12 / 170	25 / 206	0 / 173	38 / 638	13 / 492
Gof on F^2^	0.901	1.009	1.086	1.038	1.027
*R*_1_ / *wR*_2_ [*I* > 2σ(*I*))	0.0440 / 0.0867	0.0337 / 0.0638	0.0399 / 0.0828	0.0494 / 0.0991	0.0411 / 0.0732
*R*_1_ / *wR*_2_ (all data)	0.0808 / 0.0943	0.0555 / 0.0712	0.0594 / 0.0896	0.0851 / 0.1140	0.0676 / 0.0809
Residual electron density /e**·**Å^–3^	0.835 / –0.599	1.089 / –0.726	1.047 / –0.399	2.510 / –1.149	1.296 / –0.947

Two component twin; twin domain ratio 0.655(1):0.345(1).

Crystallographic data for the structures in this paper have been deposited with the Cambridge Crystallographic Data Centre, CCDC, 12 Union Road, Cambridge CB21EZ, UK. Copies of the data can be obtained free of charge on quoting the depository numbers CCDC‐1974713 (**2**), CCDC‐1974714 (**3**), CCDC‐1974716 (**4a**), CCDC‐1974717 (**4b**), and CCDC‐1974715 (**5**) (Fax: +44‐1223‐336‐033; E‐Mail: deposit@ccdc.cam.ac.uk, http://www.ccdc.cam.ac.uk).

**Supporting Information** (see footnote on the first page of this article): ^1^H, ^13^C{^1^H}, ^31^P{^1^H} NMR, IR and mass spectra (ESI(+)) of complexes **2**, **3**, **4a**, **4b** and **5**, and ^31^P{^1^H} NMR spectrum of the reaction mixture of **1a** with AgBr, ratio 3:2.

## Supporting information

Supporting information for this article is available on the WWW under https://doi.org/10.1002/zaac.202000001 or from the author.

Supporting Information
