# Supplementary material for: Silver(I) Complexes of Two Flexible Bis‐phospholane Ligands: Metallamacrocycles, Polymeric Chains, and Metallacryptands
Source: Z Anorg Allg Chem. 2020 May 12;646(13):915–22. doi: 10.1002/zaac.202000001 (PMC7386914; doi:10.1002/zaac.202000001)

Z. Anorg. Allg. Chem. **2020** · ISSN 0044–2313

SUPPORTING INFORMATION

Title: Silver(I) Complexes of Two Flexible Bis-phospholane Ligands: Metallamacrocycles, Polymeric Chains, and Metallacryptands

Author(s): P. Boar, P. Lönnecke, E. Hey-Hawkins*

Ref. No.: z202000001

Supporting Information

Silver(I) Complexes of two Flexible Bis-phospholane Ligands: Metallamacrocycles, Polymeric Chains and Metallacryptands

Paul Boar,[a] Peter Lönnecke,[a] and Evamarie Hey-Hawkins*[a]

Contents **Page**

Fig S1. $^{31}P\{^1H\}$ NMR spectrum, reaction mixture of ligand **1a** with AgBr, ratio 1:1, 1

Fig S2. $^{31}P\{^1H\}$ NMR spectrum, reaction mixture of ligand **1a** with AgBr, ratio 3:2 2

Fig S3-1. ^1H NMR spectrum of complex **2** in $CDCl_3$ 3

Fig S3-2. $^{13}C\{^1H\}$ NMR spectrum of complex **2** in $CDCl_3$ (inset: enlarged section) 4

Fig S3-3. $^{31}P\{^1H\}$ NMR spectrum of complex **2** in $CDCl_3$ 5

Fig S3-4. MS (ESI(+), DCM/Acetonitrile) of complex **2** 6

Fig S3-5. IR spectrum of complex **2** 7

Fig S4-1. ^1H NMR spectrum of complex **3** in $CDCl_3$ 8

Fig S4-2. $^{13}C\{^1H\}$ NMR spectrum of complex **3** in $CDCl_3$ (inset: enlarged section) 9

Fig S4-3. $^{31}P\{^1H\}$ NMR spectrum of complex **3** in $CDCl_3$ 10

Fig S4-4. MS (ESI(+), DCM/Acetonitrile) of complex **3** 11

Fig S4-5. IR spectrum of complex **3** 12

Fig S5-1. ^1H NMR spectrum of complex **4a** in $CDCl_3$ 13

Fig S5-2. $^{13}C\{^1H\}$ NMR spectrum of complex **4a** in $CDCl_3$ (inset: enlarged section) 14

Fig S5-3. $^{31}P\{^1H\}$ NMR spectrum of complex **4a** in $CDCl_3$ 15

Fig S5-4. MS (ESI(+), DCM/MeOH) of complex **4a** 16

Fig S5-5. IR spectrum of complex **4a** 17

---

[a] Dr. P. Boar, Dr. P. Lönnecke, Prof. Dr. E. Hey-Hawkins*
Faculty of Chemistry and Mineralogy
Institute of Inorganic Chemistry
Johannisallee 29
D-04103 Leipzig
Germany
E-mail: hey@uni-leipzig.de

Fig S6-1. ^{1}H NMR spectrum of complex **4b** in $CDCl_3$ 18

Fig S6-2. $^{13}C\{^{1}H\}$ NMR spectrum of complex **4b** in $CDCl_3$ (inset: enlarged section) 19

Fig S6-3. $^{31}P\{^{1}H\}$ NMR spectrum of complex **4b** in $CDCl_3$ 20

Fig S6-4. MS (ESI(+), DCM/MeOH) of complex **4b** 21

Fig S6-5. IR spectrum of complex **4b** 22

Fig S7-1. ^{1}H NMR spectrum of complex **5** in $CDCl_3$ 23

Fig S7-2. $^{13}C\{^{1}H\}$ NMR spectrum of complex **5** in $CDCl_3$ (inset: enlarged section) 24

Fig S7-3. $^{31}P\{^{1}H\}$ NMR spectrum of complex **5** in $CDCl_3$ 25

Fig S7-4. MS (ESI(+), DCM/MeOH) of complex **5** 26

Fig S7-5. IR spectrum of complex **5** 27

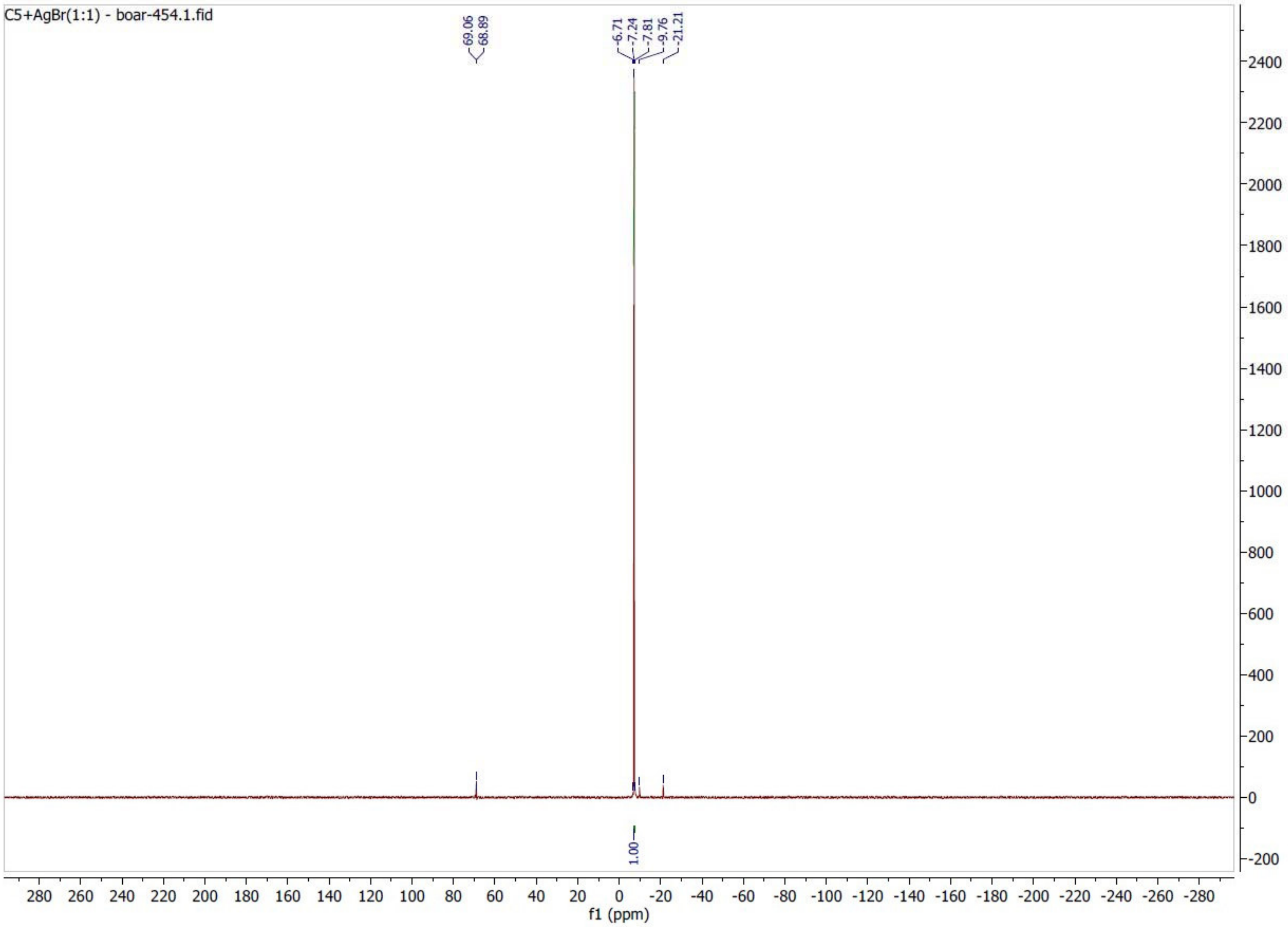

Fig S1. $^{31}P\{^{1}H\}$ NMR spectrum, reaction mixture of ligand **1a** with AgBr, ratio 1:1,

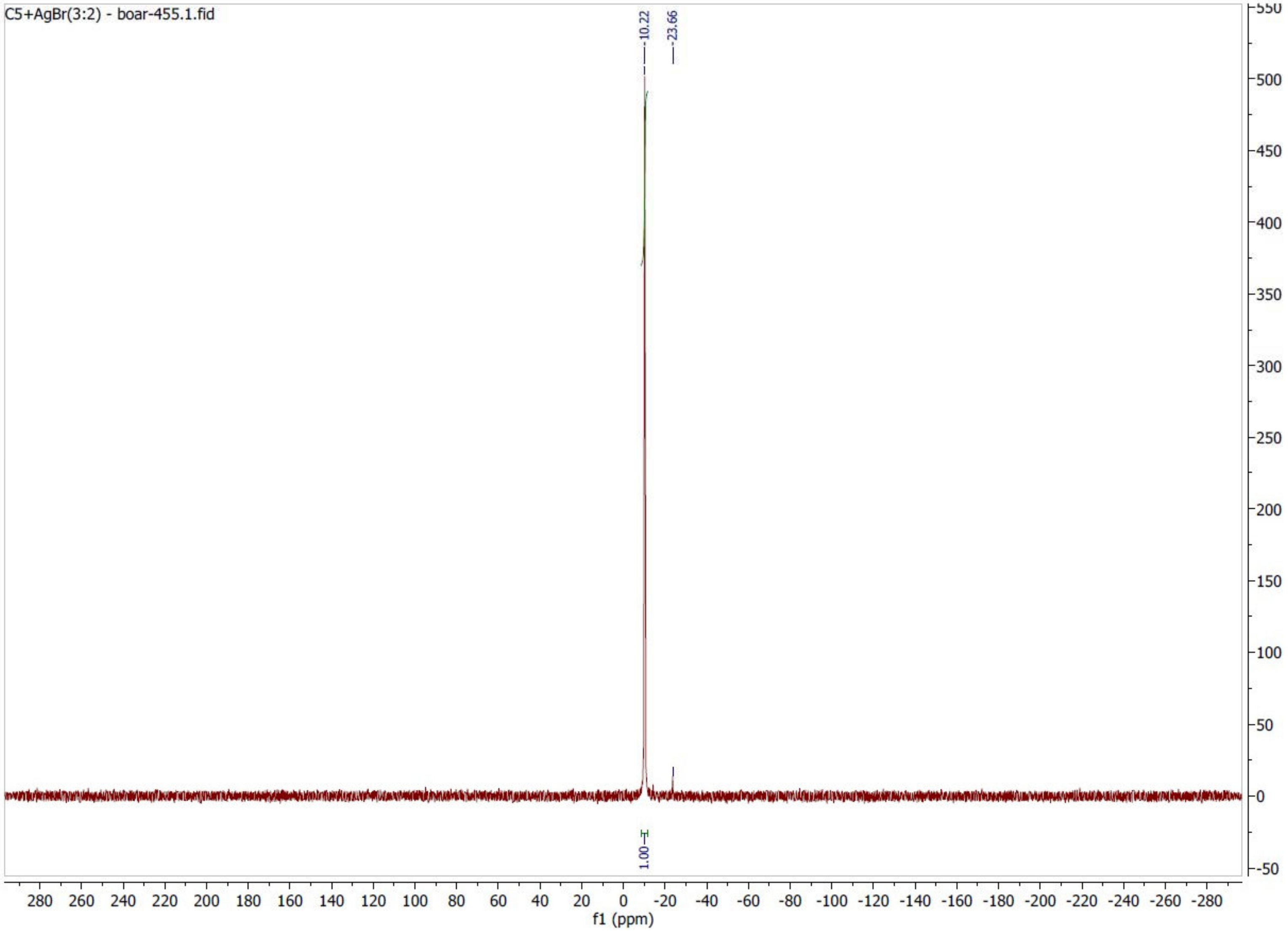

Fig S2. $^{31}P\{^{1}H\}$ NMR spectrum, reaction mixture of ligand **1a** with AgBr, ratio 3:2

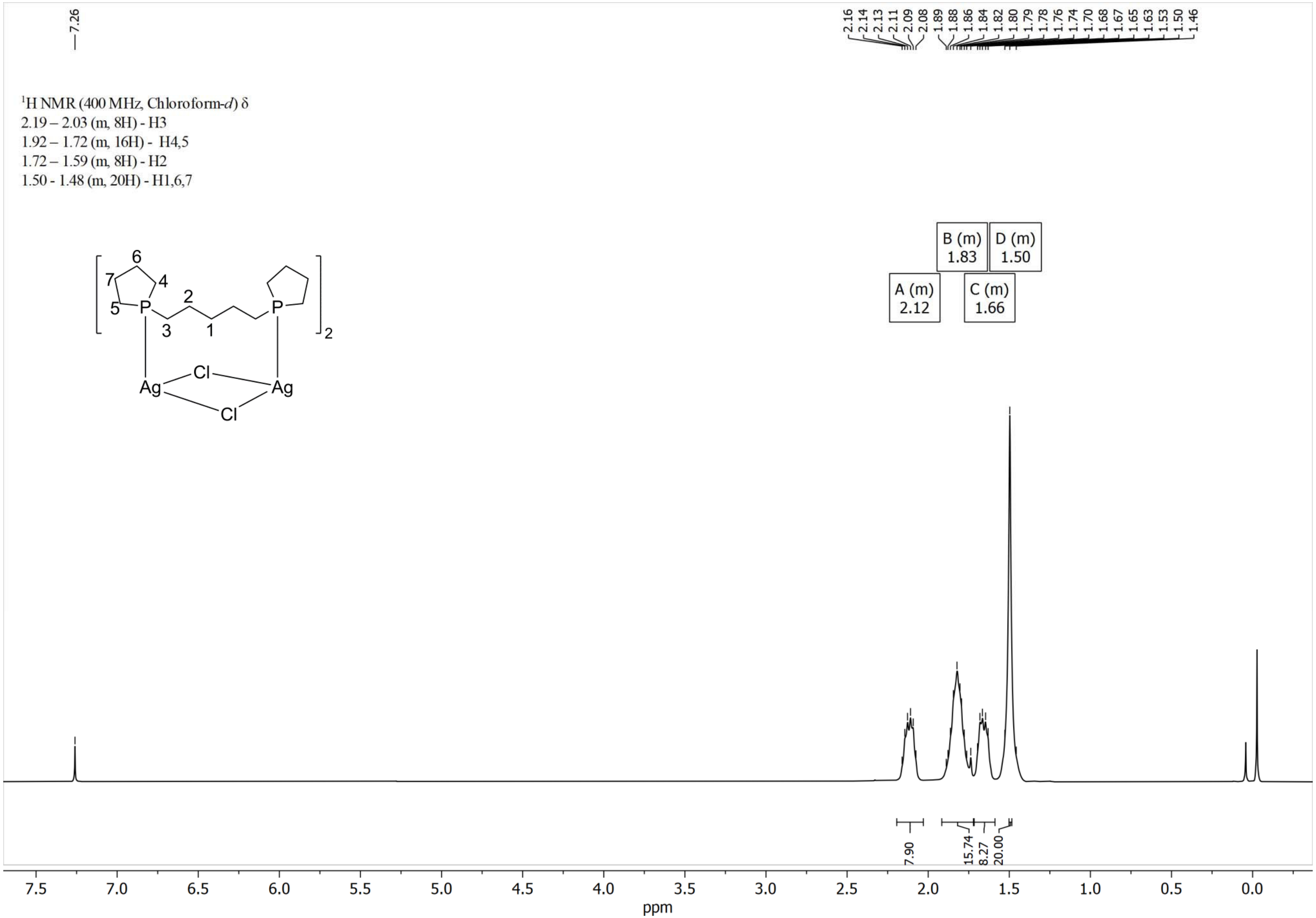

Fig S3-1. ^{1}H NMR spectrum of complex **2** in $CDCl_3$

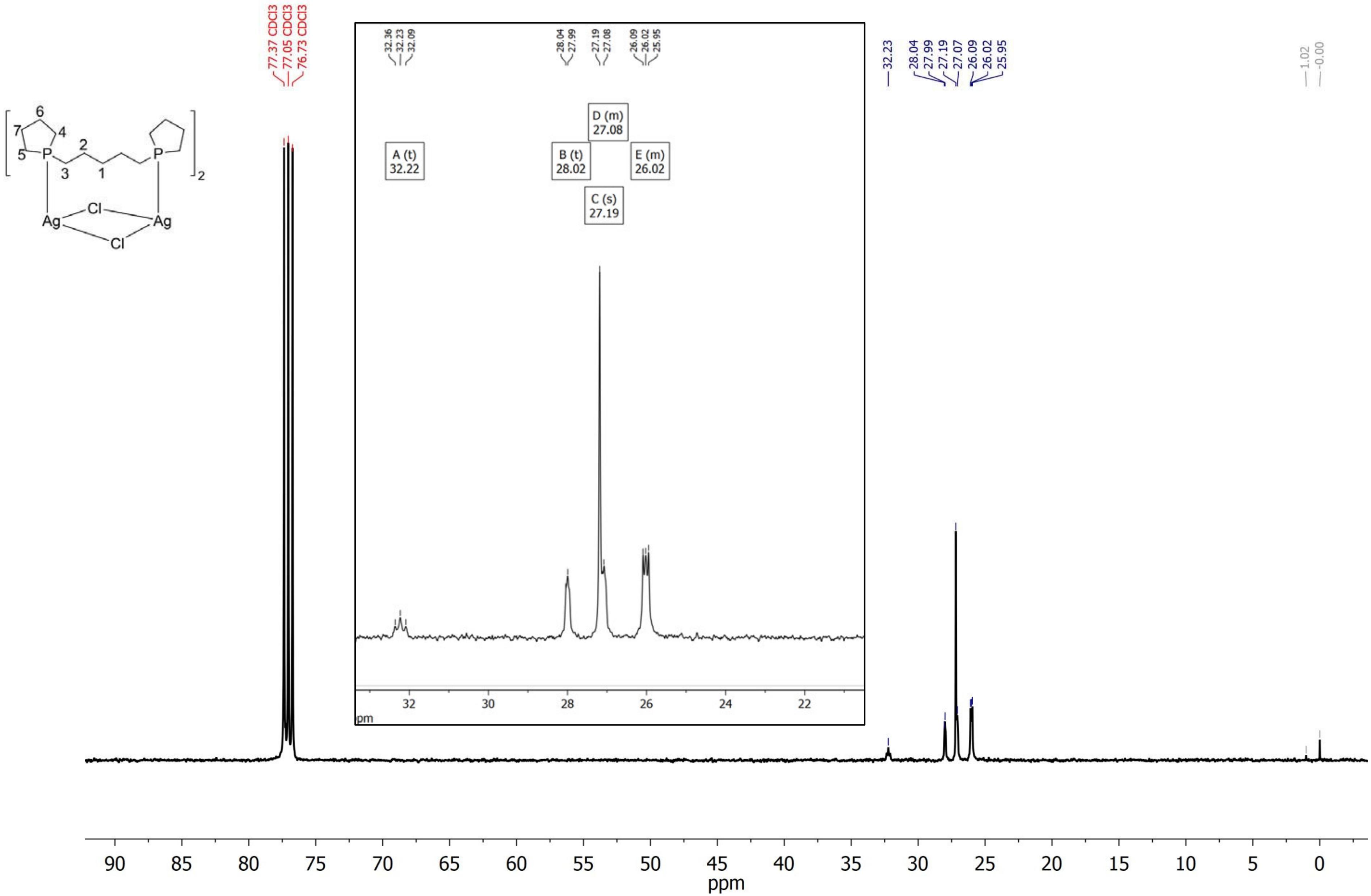

Fig S3-2. $^{13}C\{^{1}H\}$ NMR spectrum of complex **2** in $CDCl_3$ (inset: enlarged section)

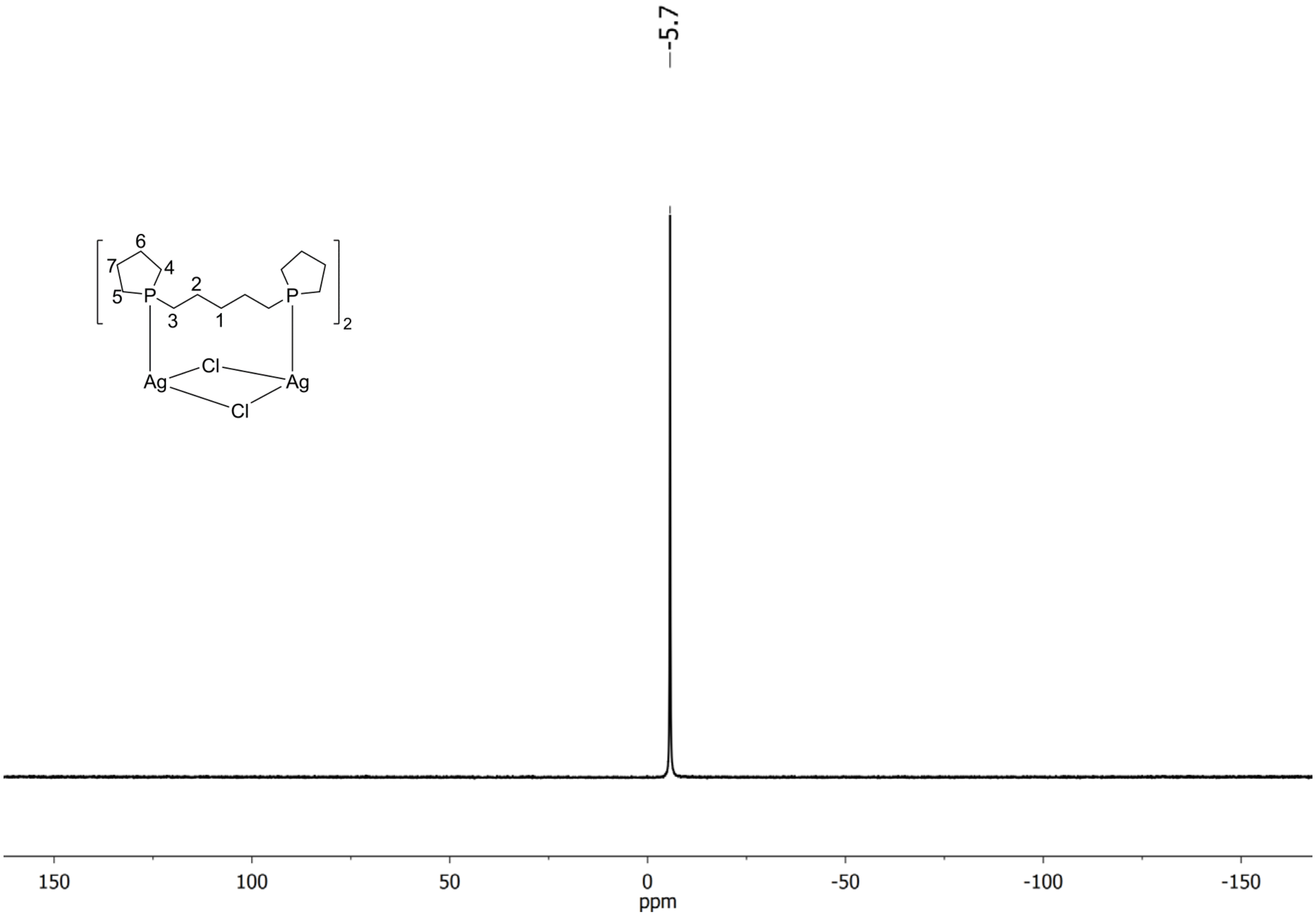

Fig S3-3. $^{31}P\{^{1}H\}$ NMR spectrum of complex **2** in $CDCl_3$

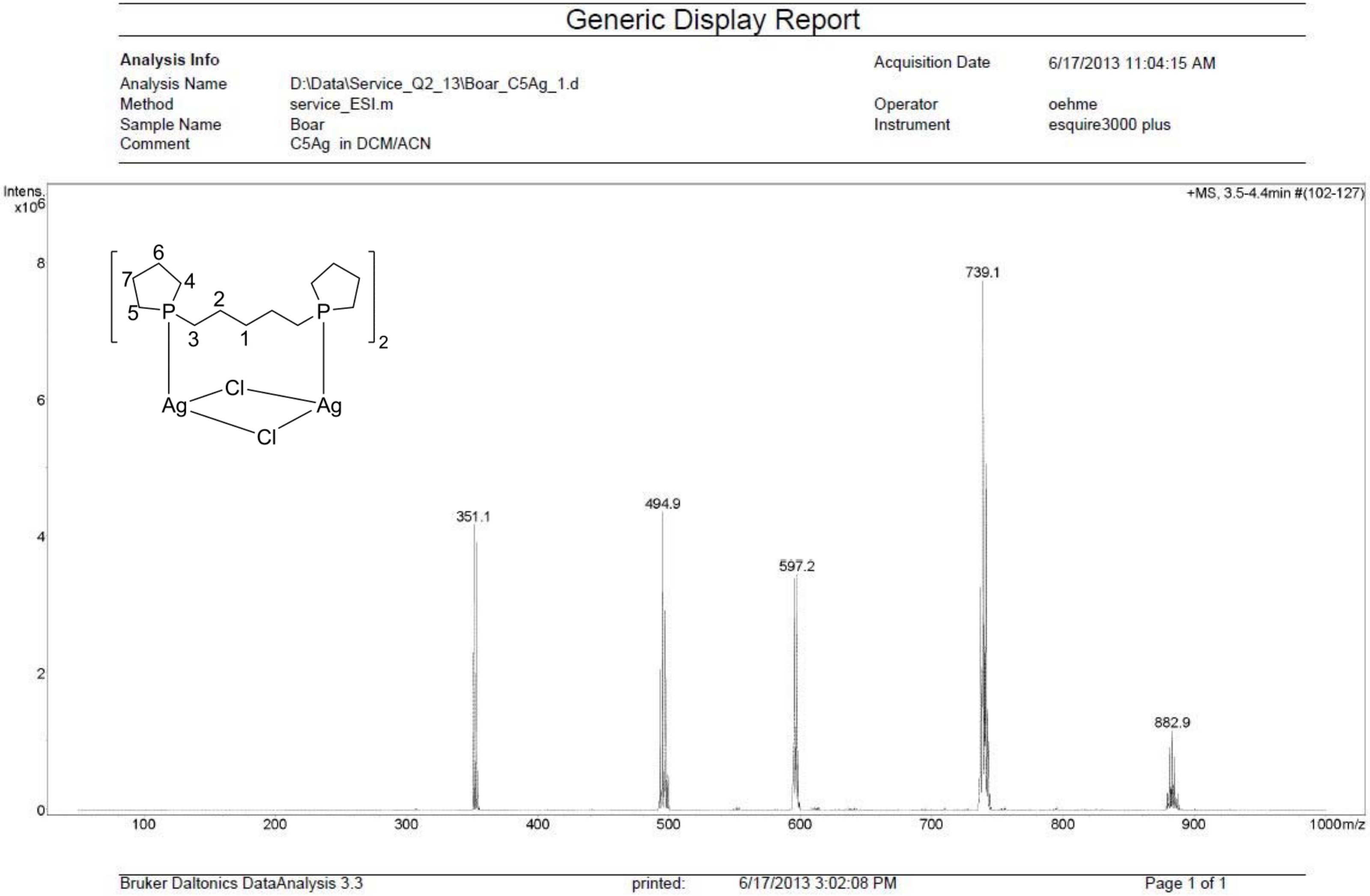

Fig S3-4. MS (ESI(+), DCM/Acetonitrile) of complex **2**

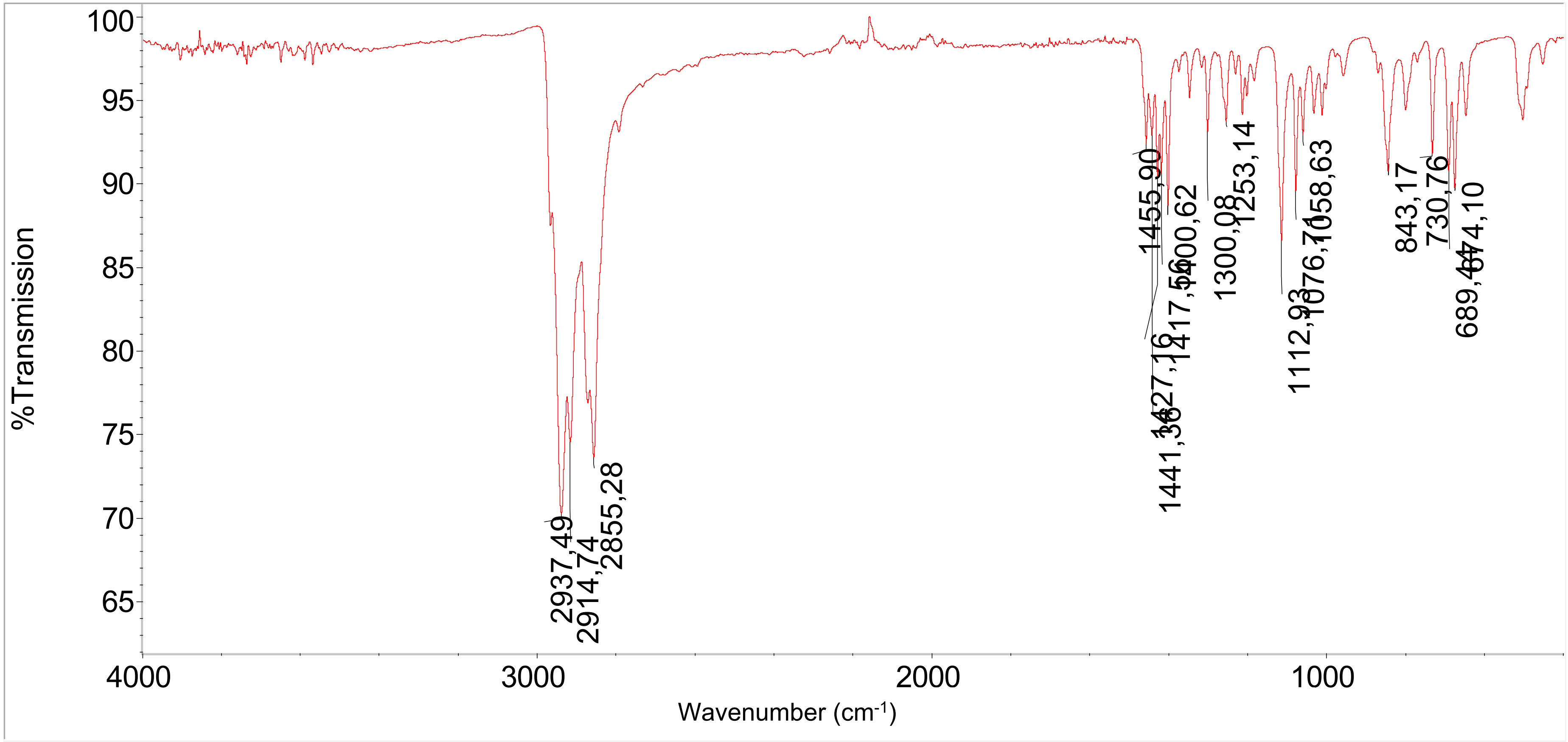

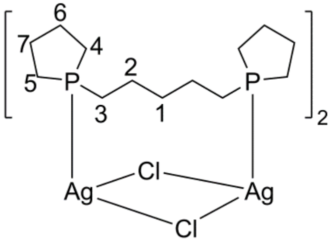

Fig S3-5. IR spectrum of complex **2**

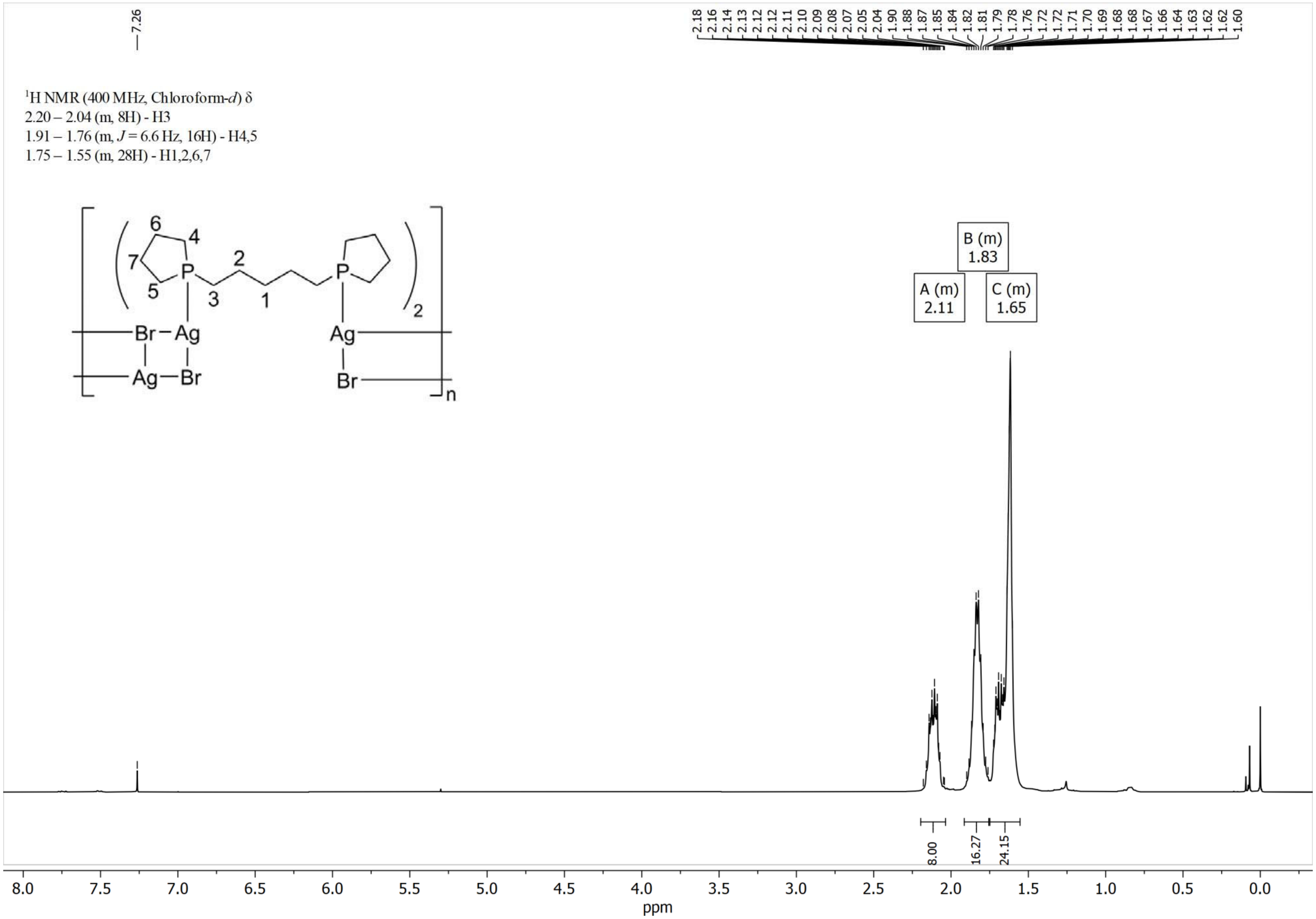

Fig S4-1. ^{1}H NMR spectrum of complex **3** in $CDCl_3$

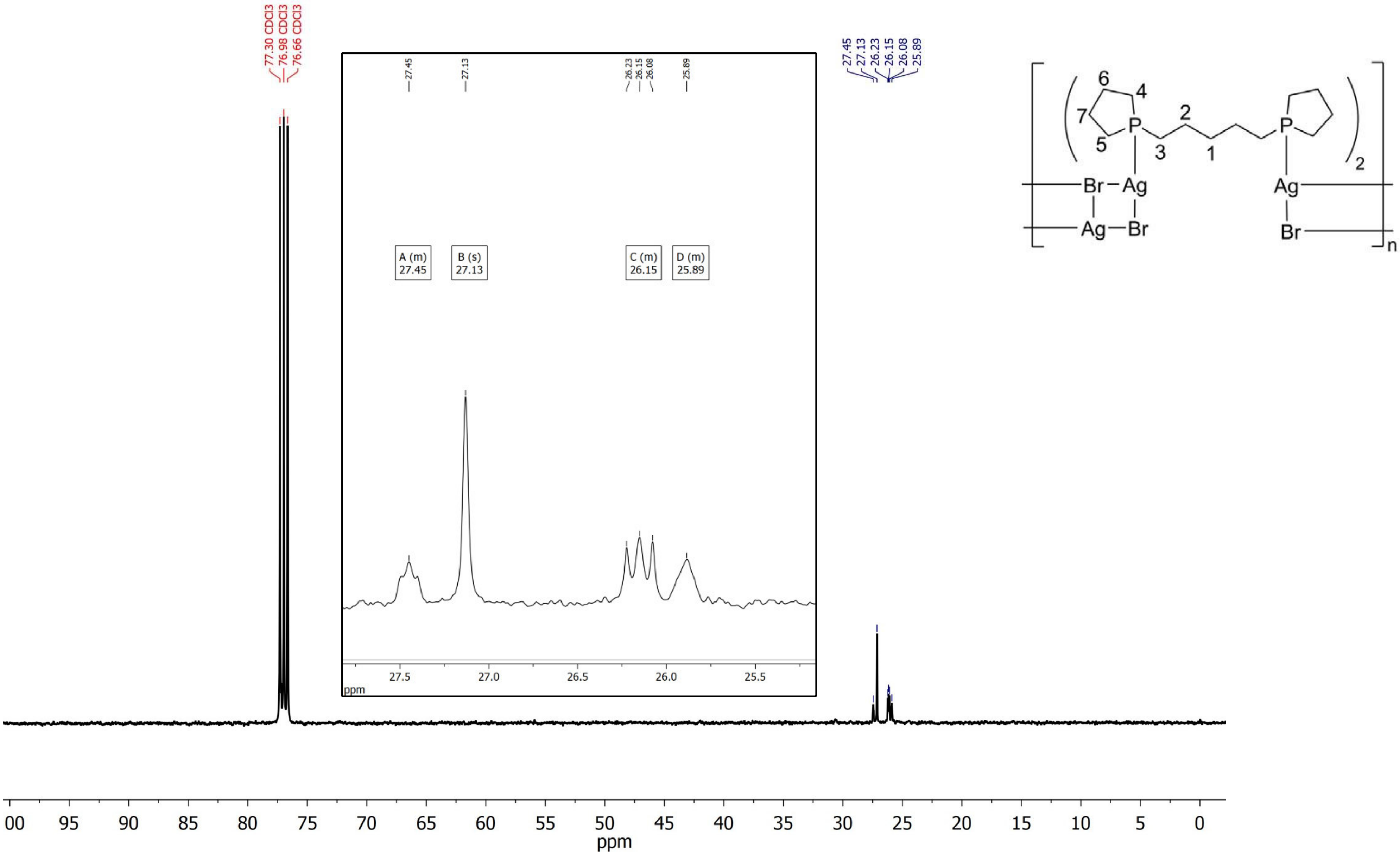

Fig S4-2. $^{13}C\{^{1}H\}$ NMR spectrum of complex **3** in $CDCl_3$ (inset: enlarged section).

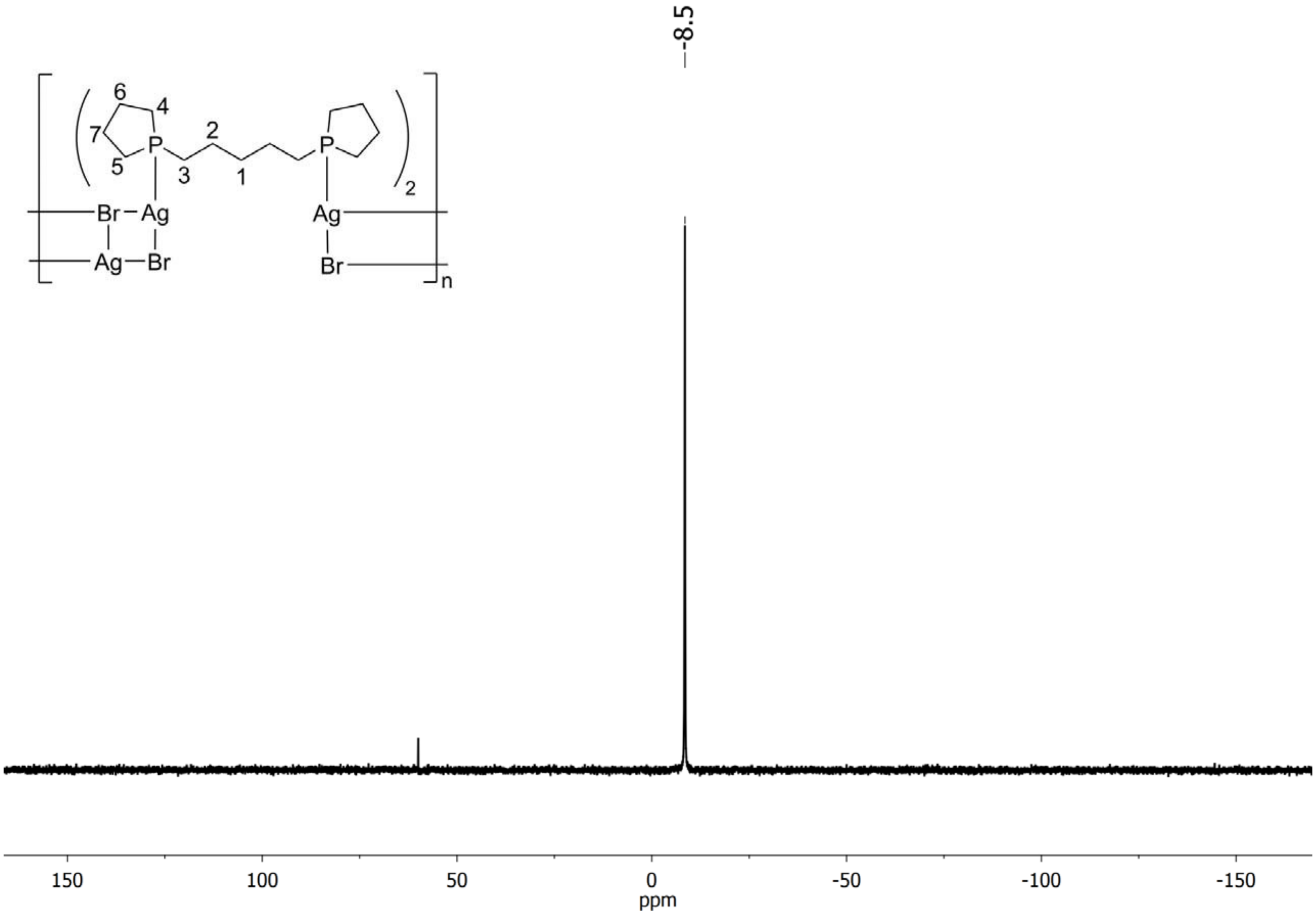

Fig S4-3. $^{31}P\{^{1}H\}$ NMR spectrum of complex **3** in $CDCl_3$

Generic Display Report

Analysis Info

Analysis Name	D:\Data\Service_Q3_16\Boar_C5AgBr1to1_1.d	Acquisition Date	7/14/2016 4:11:52 PM
Method	Service_ESI.m	Operator	oe
Sample Name	C5AgBr1to1	Instrument	impact II
Comment	in DCM/ACN		

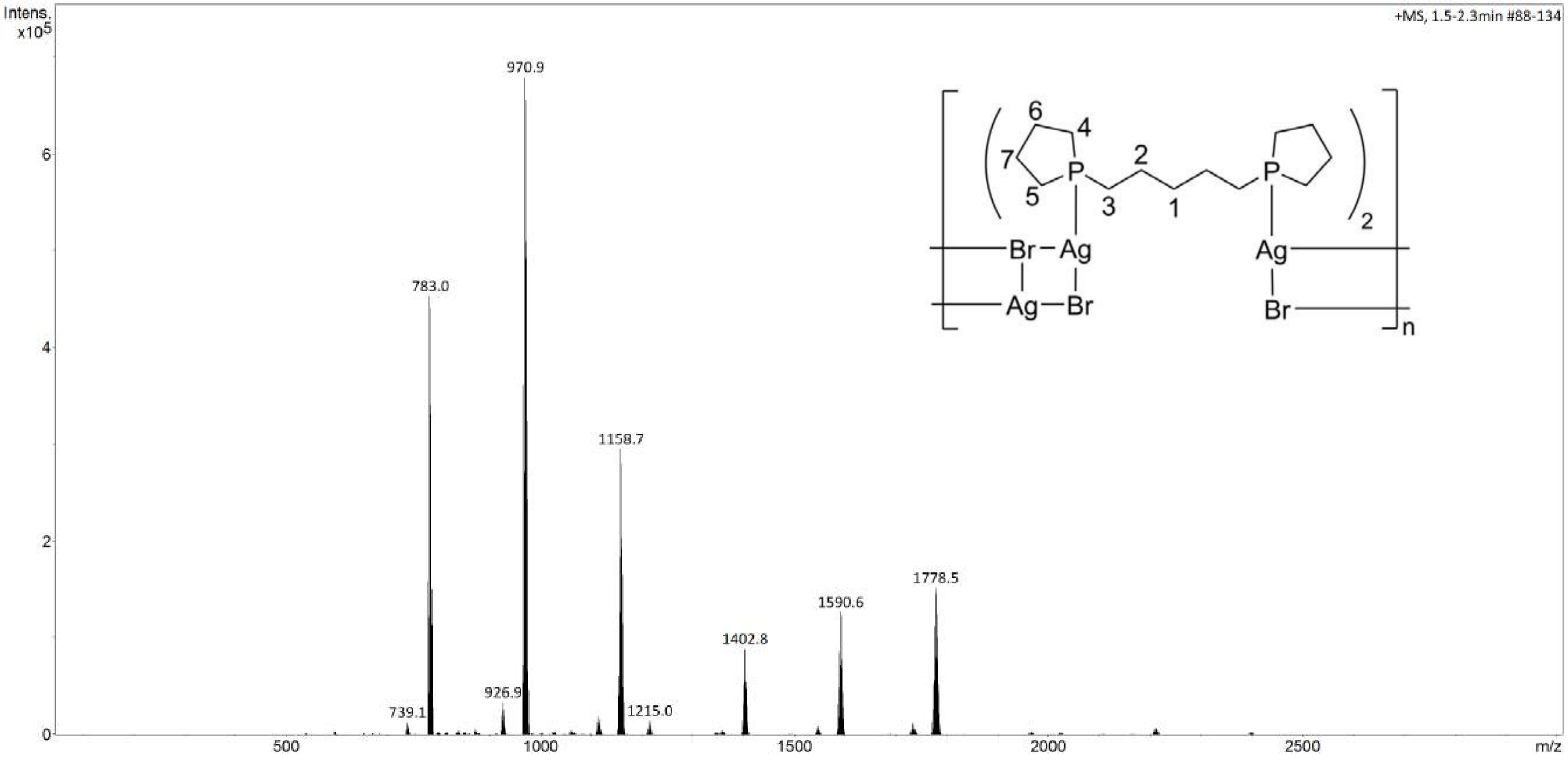

Bruker Compass DataAnalysis 4.2 printed: 7/14/2016 4:21:56 PM Page 1 of 1

Fig S4-4. MS (ESI(+), DCM/Acetonitrile) of complex **3**

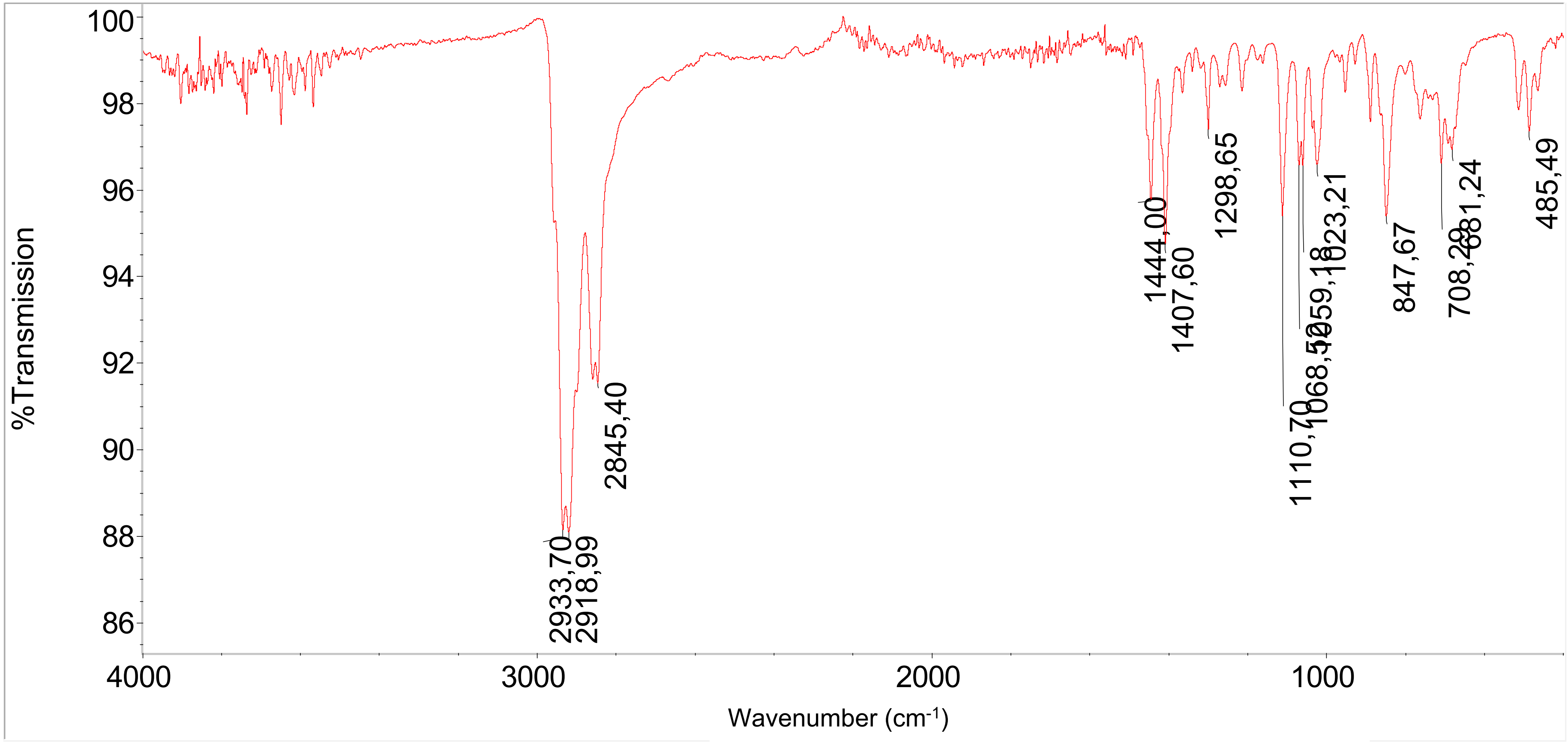

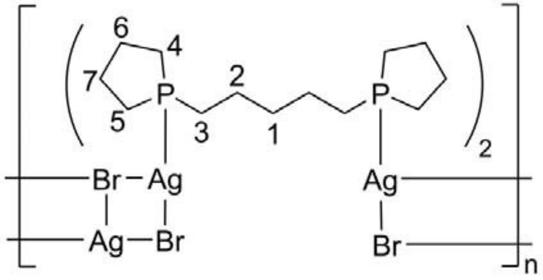

Fig S4-5. IR spectrum of complex **3**

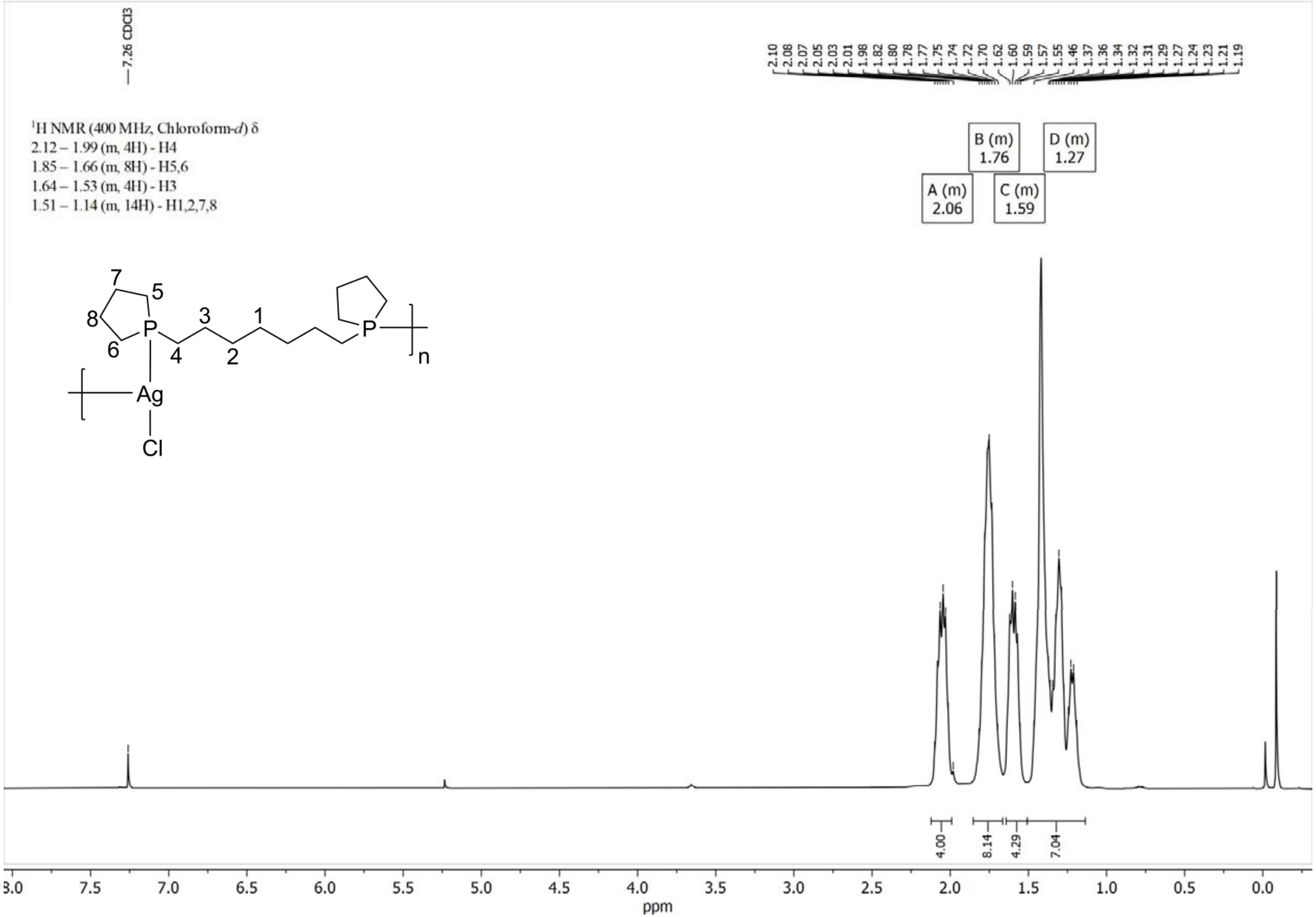

Fig S5-1. ^{1}H NMR spectrum of complex **4a** in $CDCl_3$

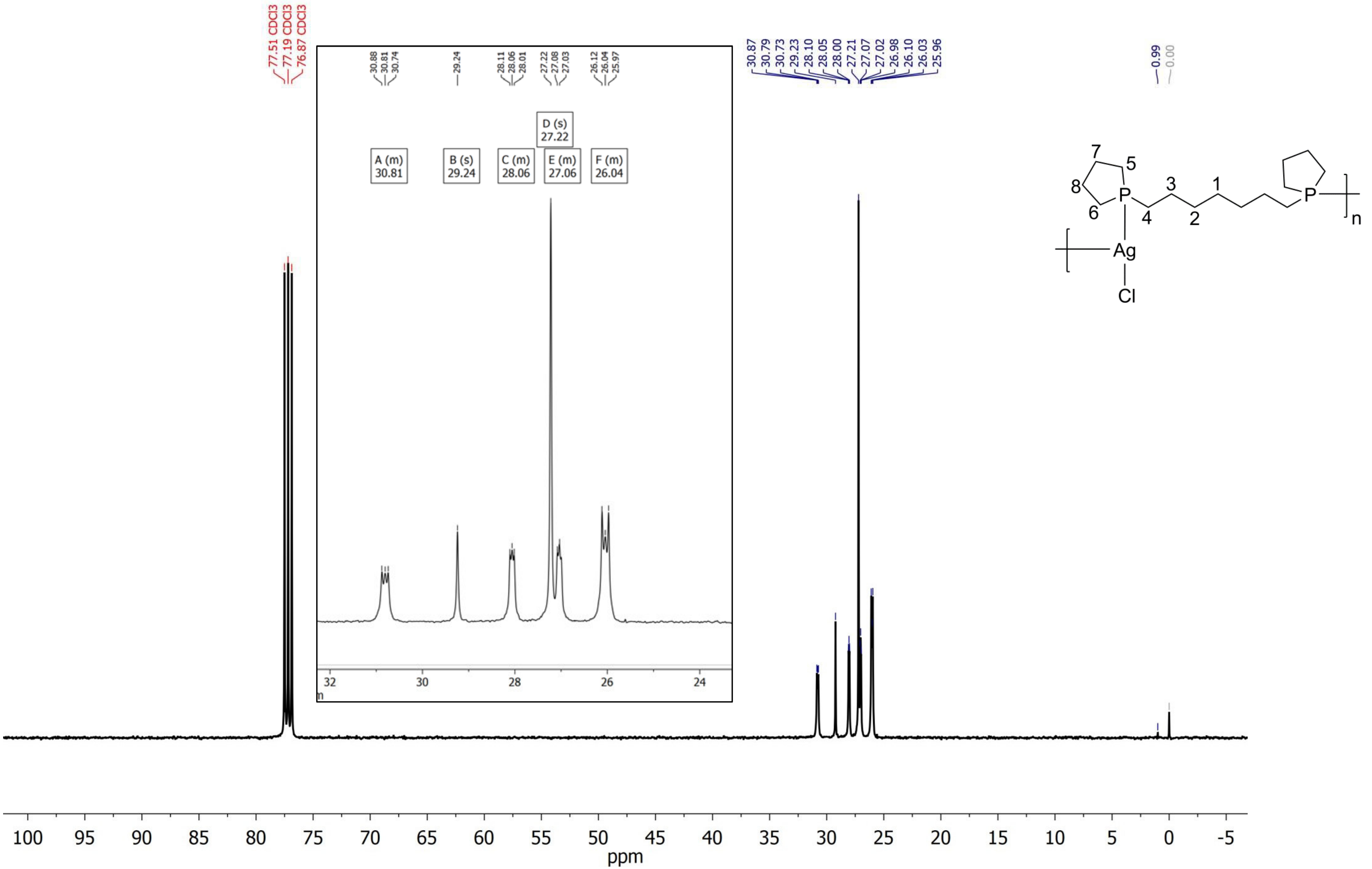

Fig S5-2. $^{13}C\{^{1}H\}$ NMR spectrum of complex **4a** in $CDCl_3$ (inset: enlarged section).

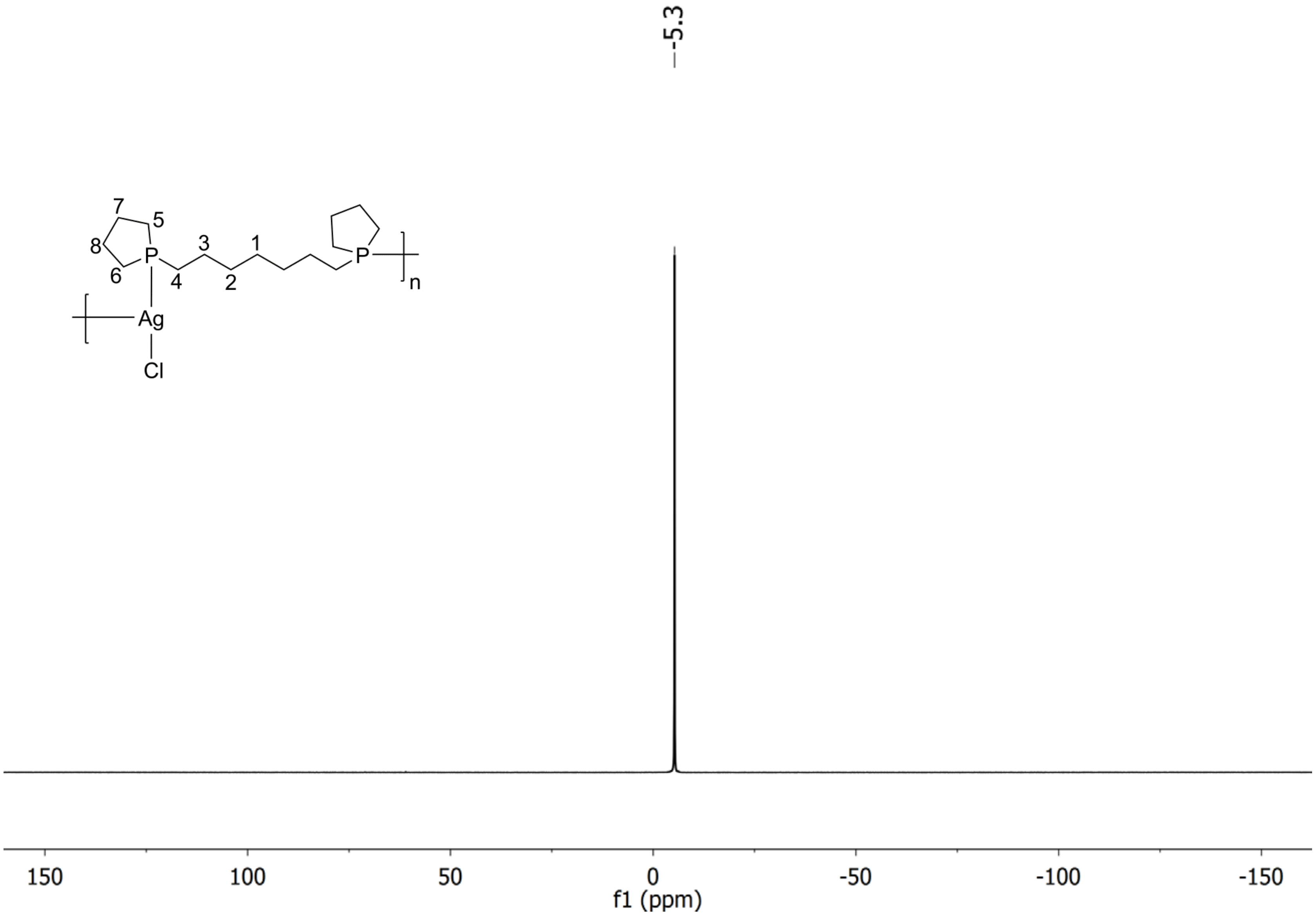

Fig S5-3. $^{31}P\{^{1}H\}$ NMR spectrum of complex **4a** in $CDCl_3$

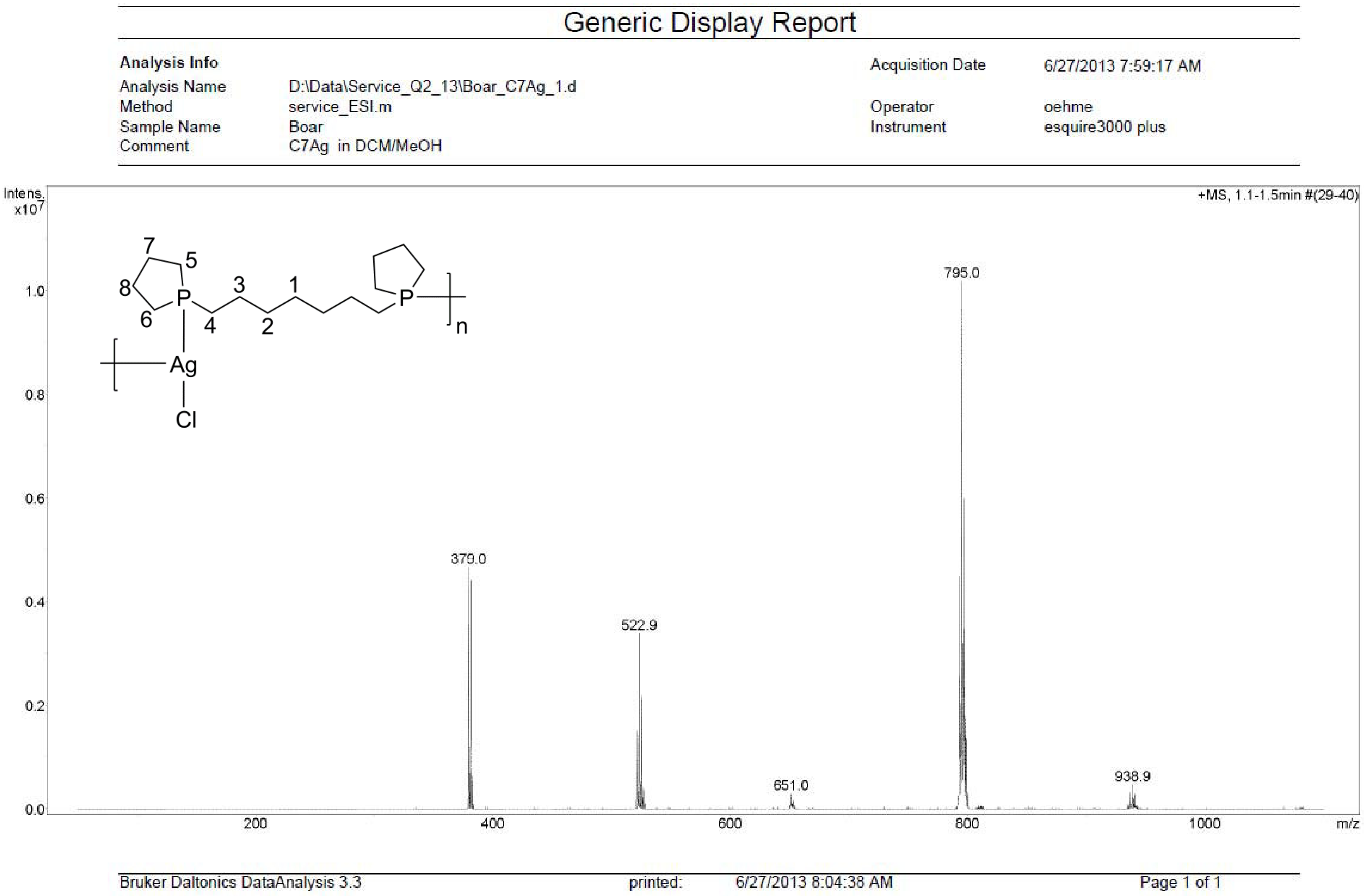

Fig S5-4. MS (ESI(+), DCM/MeOH) of complex **4a**

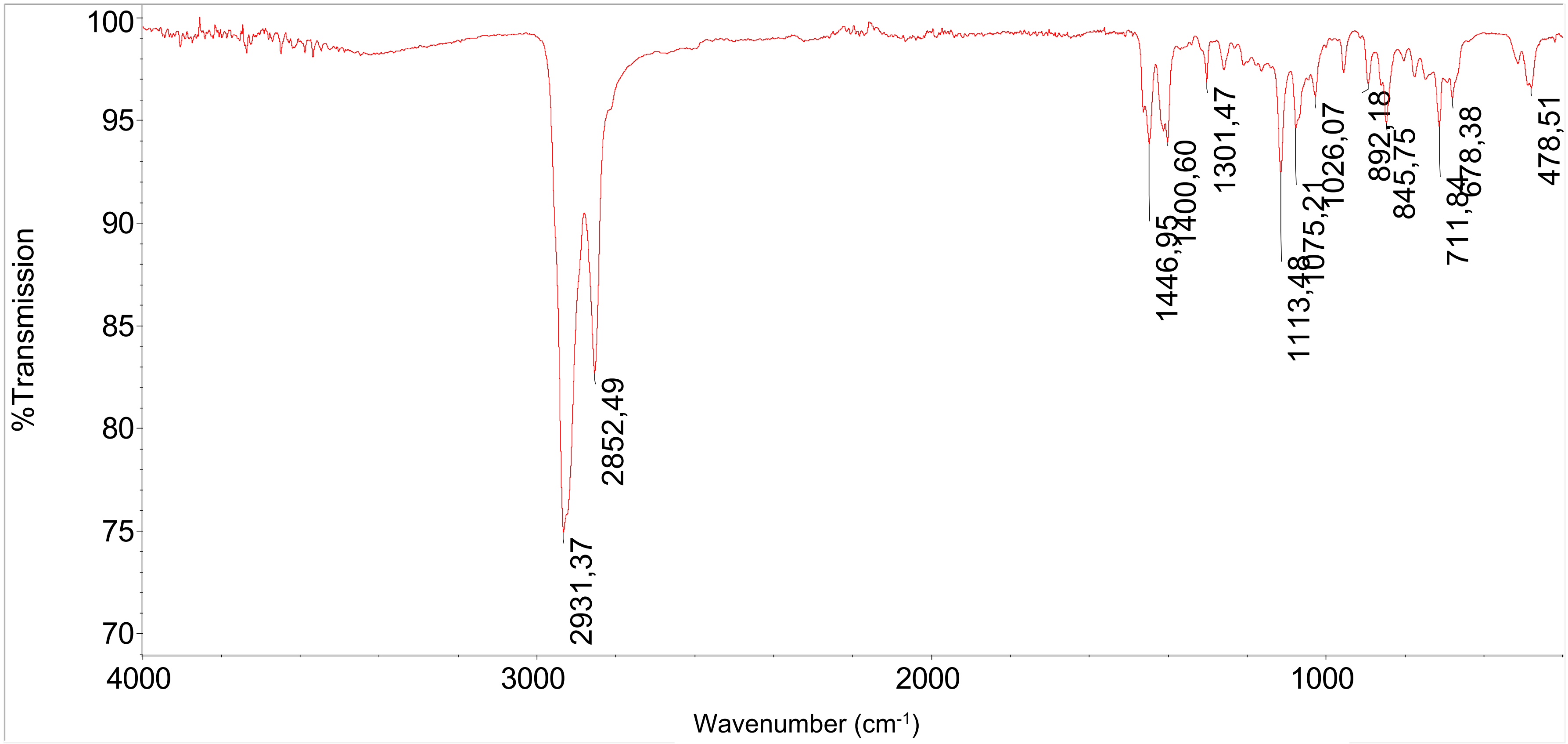

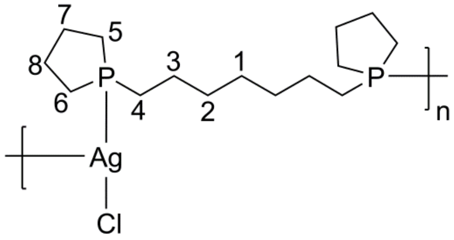

Fig S5-5. IR spectrum of complex **4a**

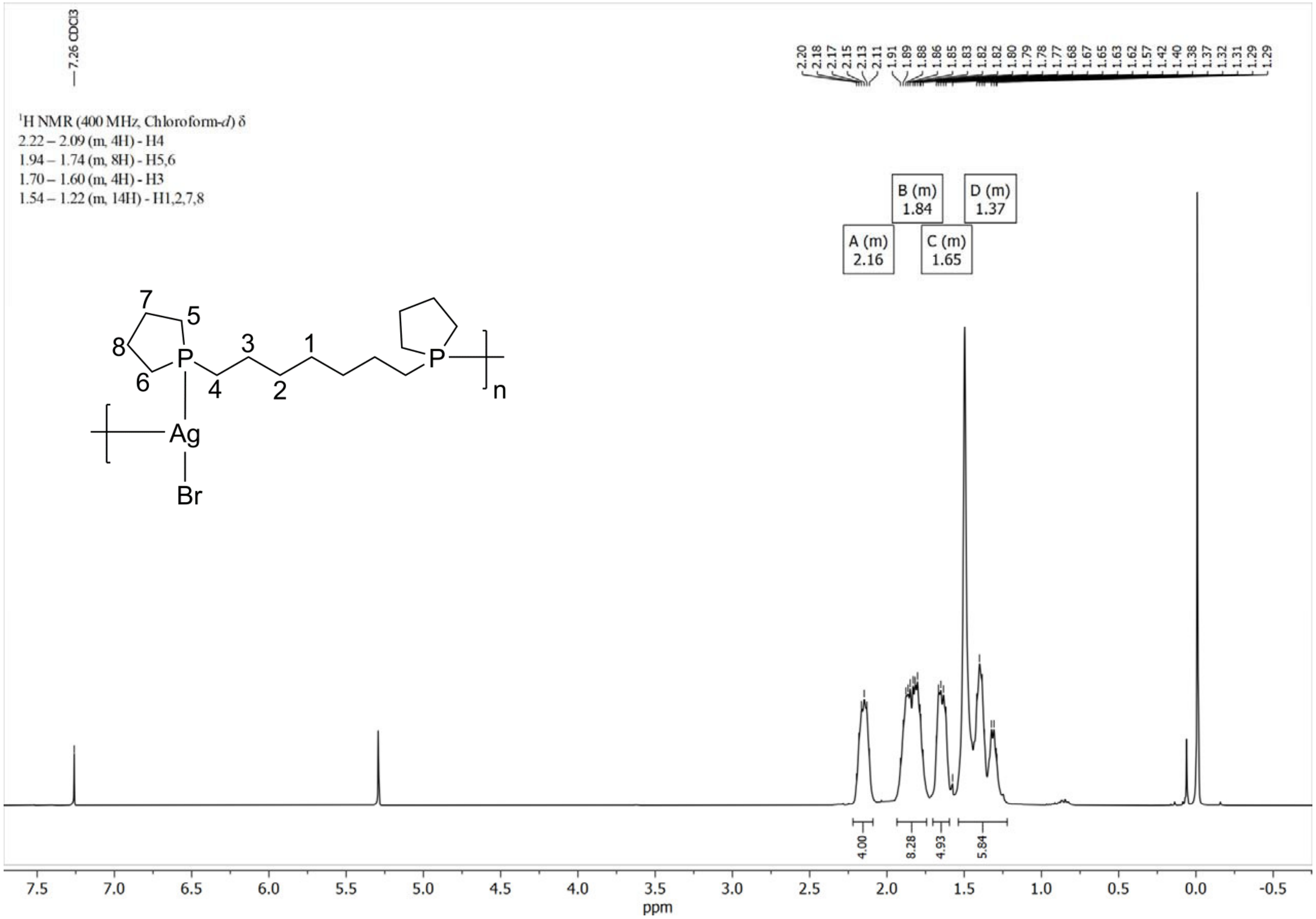

Fig S6-1. ^{1}H NMR spectrum of complex **4b** in $CDCl_3$

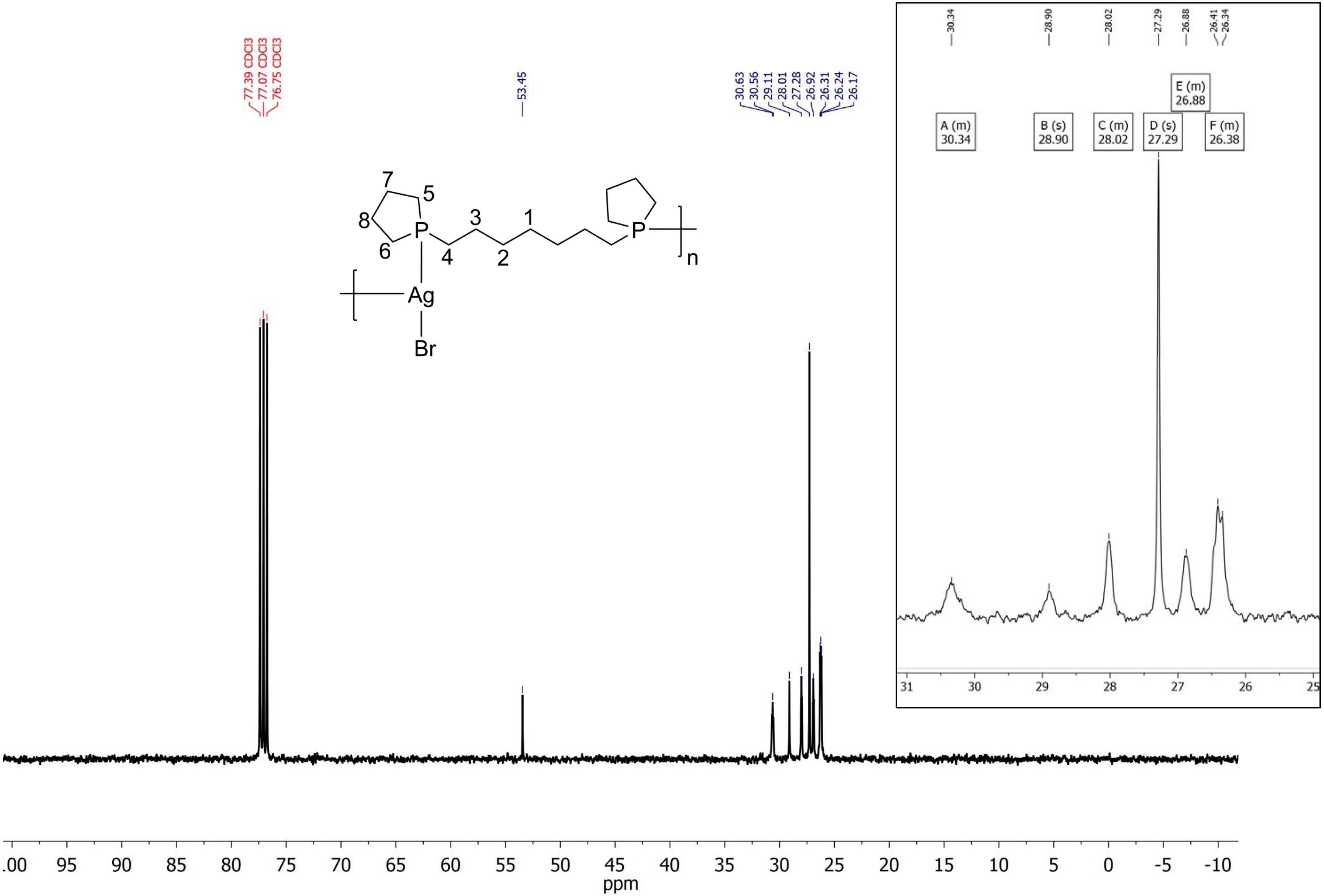

Fig S6-2. $^{13}C\{^{1}H\}$ NMR spectrum of complex **4b** in $CDCl_3$ (inset: enlarged section).

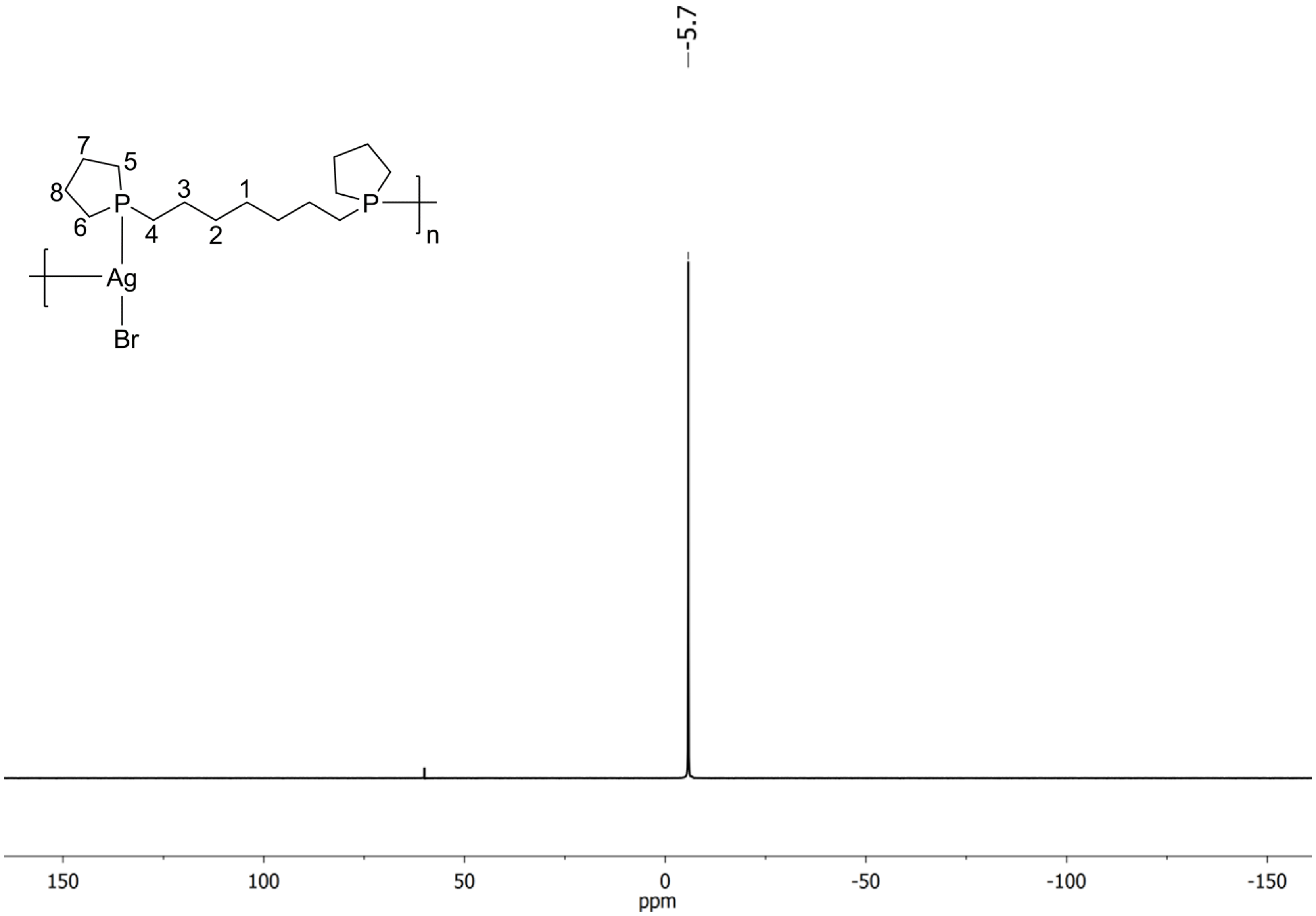

Fig S6-3. $^{31}P\{^{1}H\}$ NMR spectrum of complex **4b** in $CDCl_3$

Generic Display Report

Analysis Info		Acquisition Date	5/2/2016 9:30:58 AM
Analysis Name	D:\Data\Service_Q2_16\Boar_C7_AgBr_3_2_1.d		
Method	Service_ESI.m	Operator	oe
Sample Name	C7+AgBr (3:2)	Instrument	impact II
Comment	in DCM/MeOH		

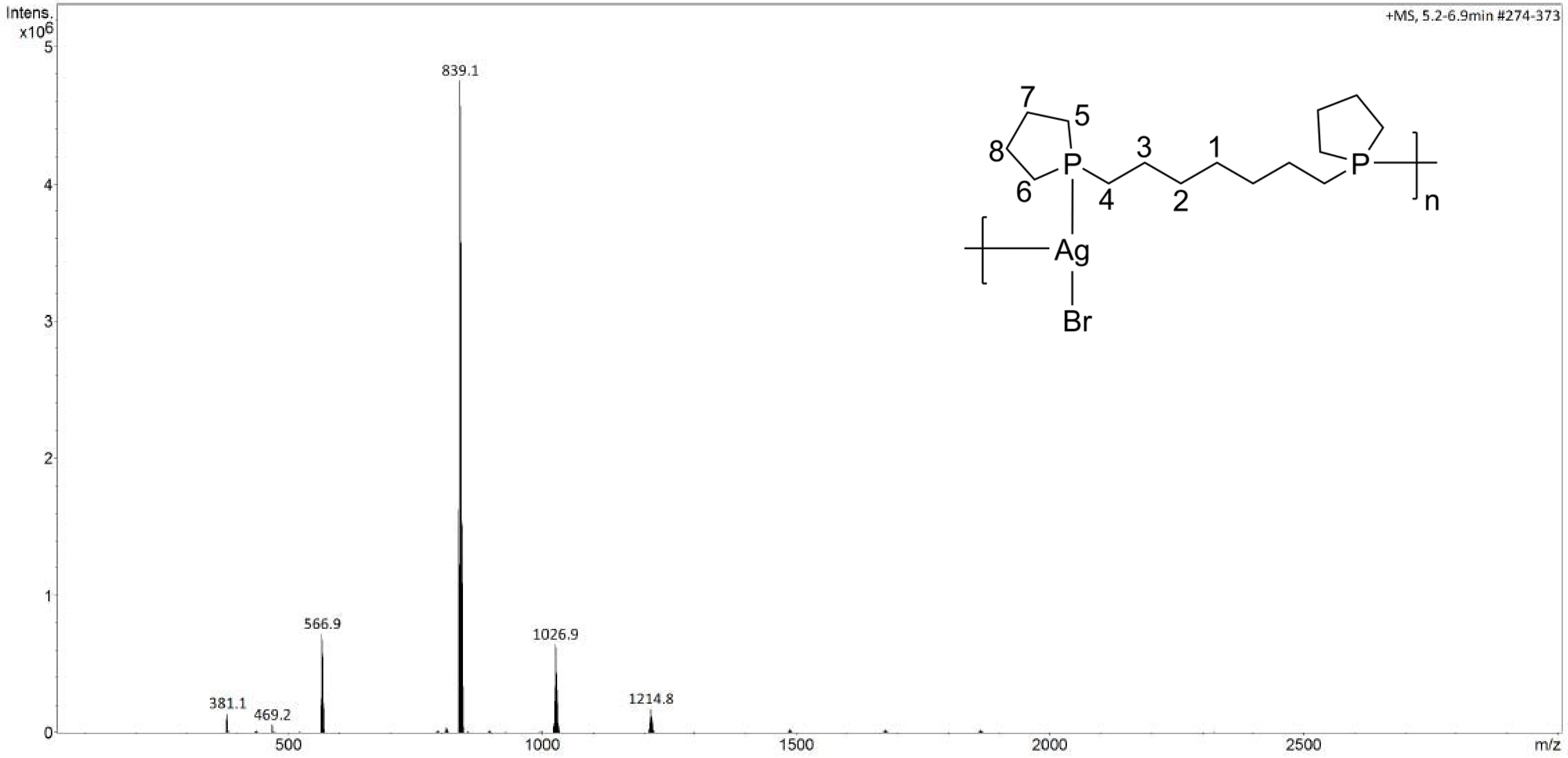

Bruker Compass DataAnalysis 4.2 printed: 5/2/2016 9:49:16 AM Page 1 of 1

Fig S6-4. MS (ESI(+), DCM/MeOH) of complex **4b**

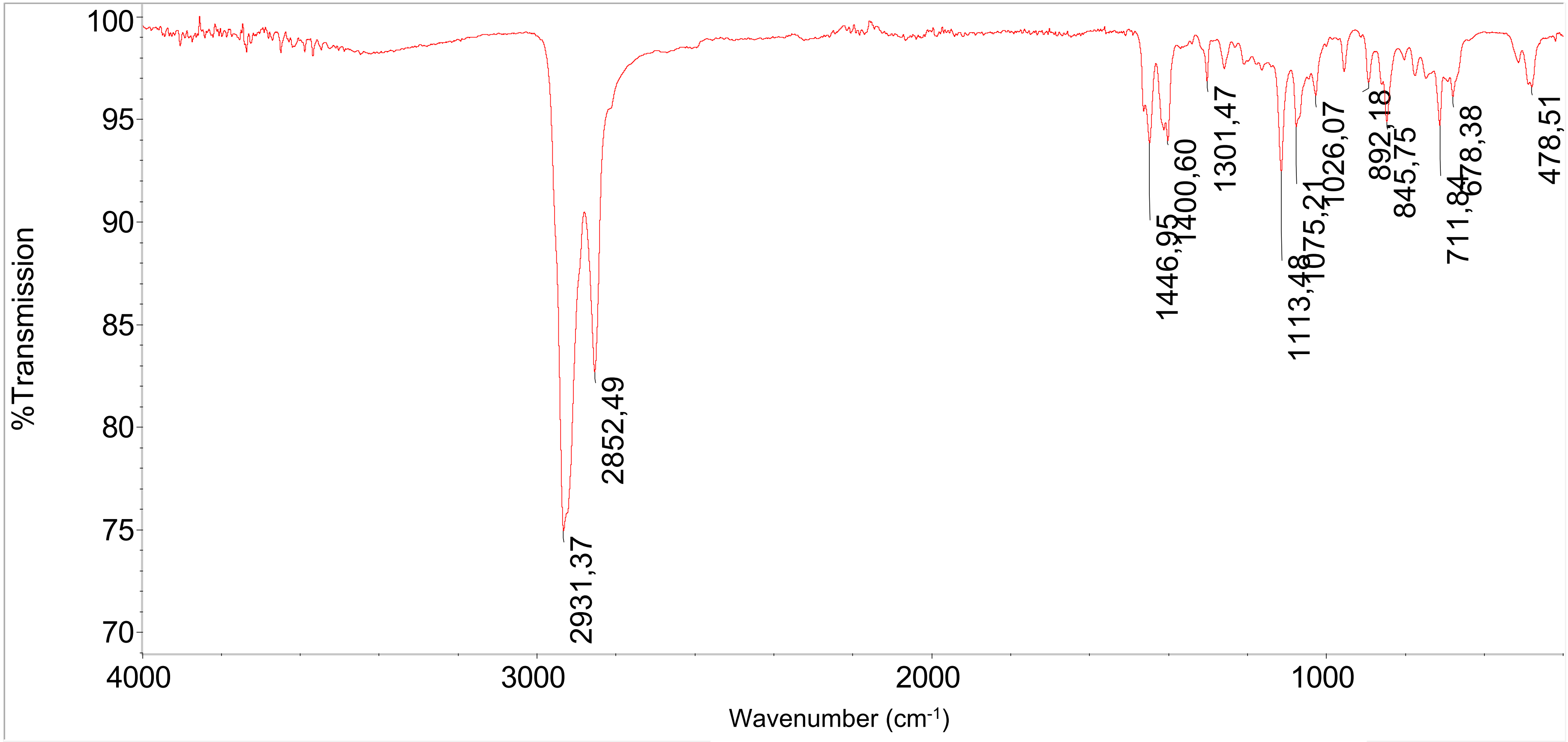

Fig S6-5. IR spectrum of complex **4b**

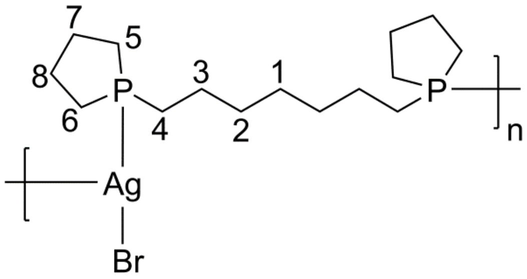

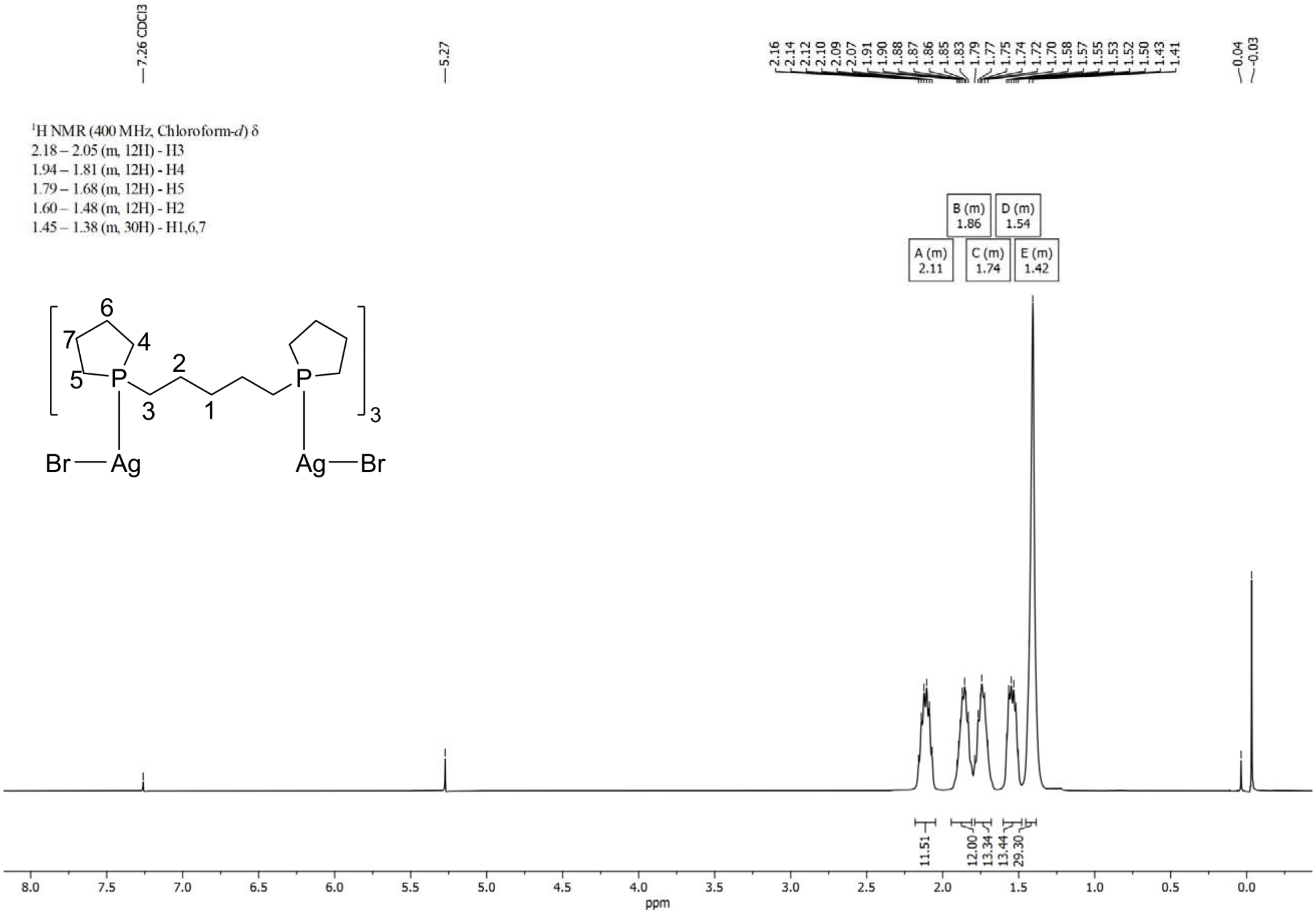

Fig S7-1. ^{1}H NMR spectrum of complex **5** in $CDCl_3$

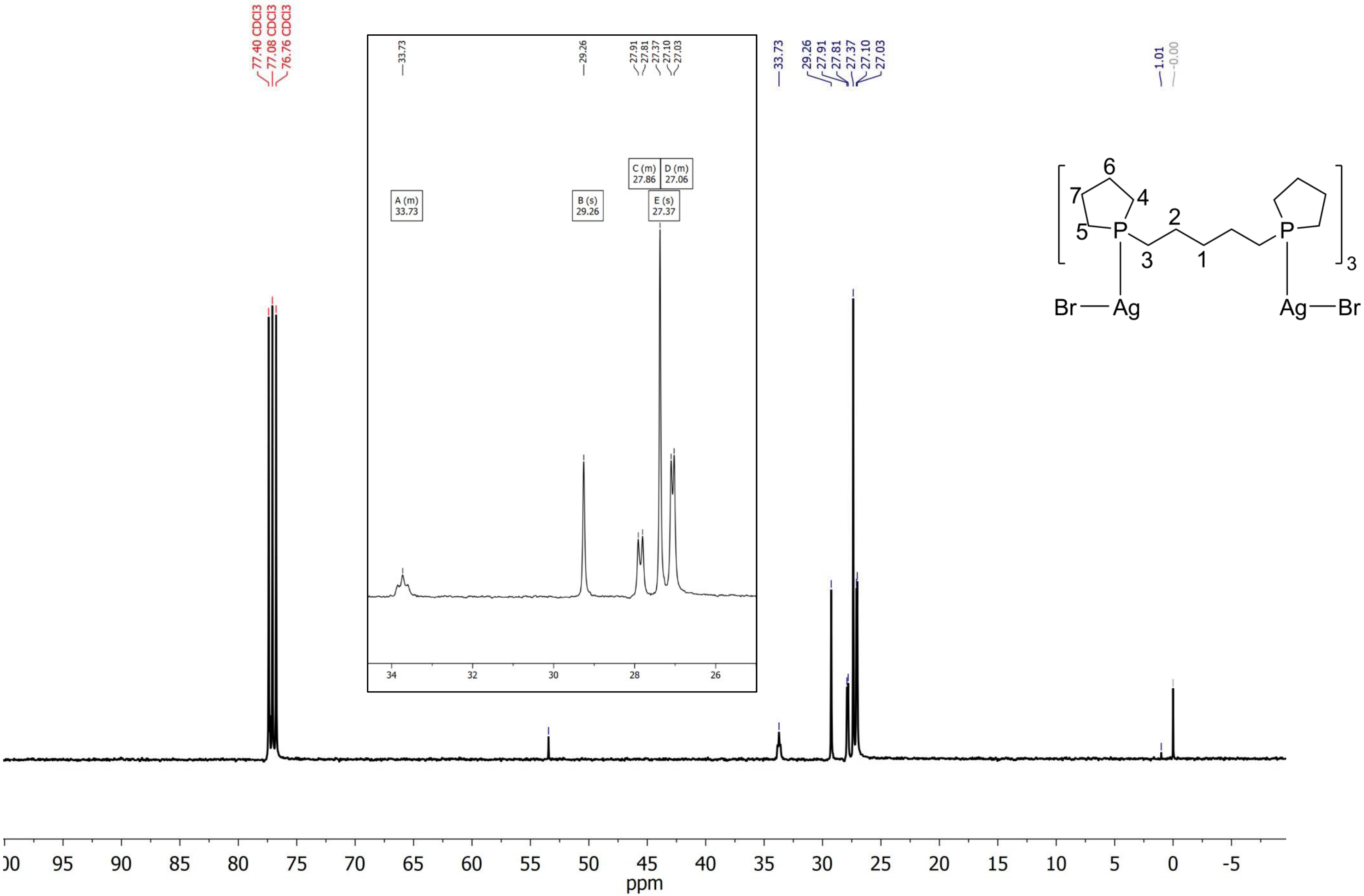

Fig S7-2. $^{13}C\{^1H\}$ NMR spectrum of complex **5** in $CDCl_3$ (inset: enlarged section)

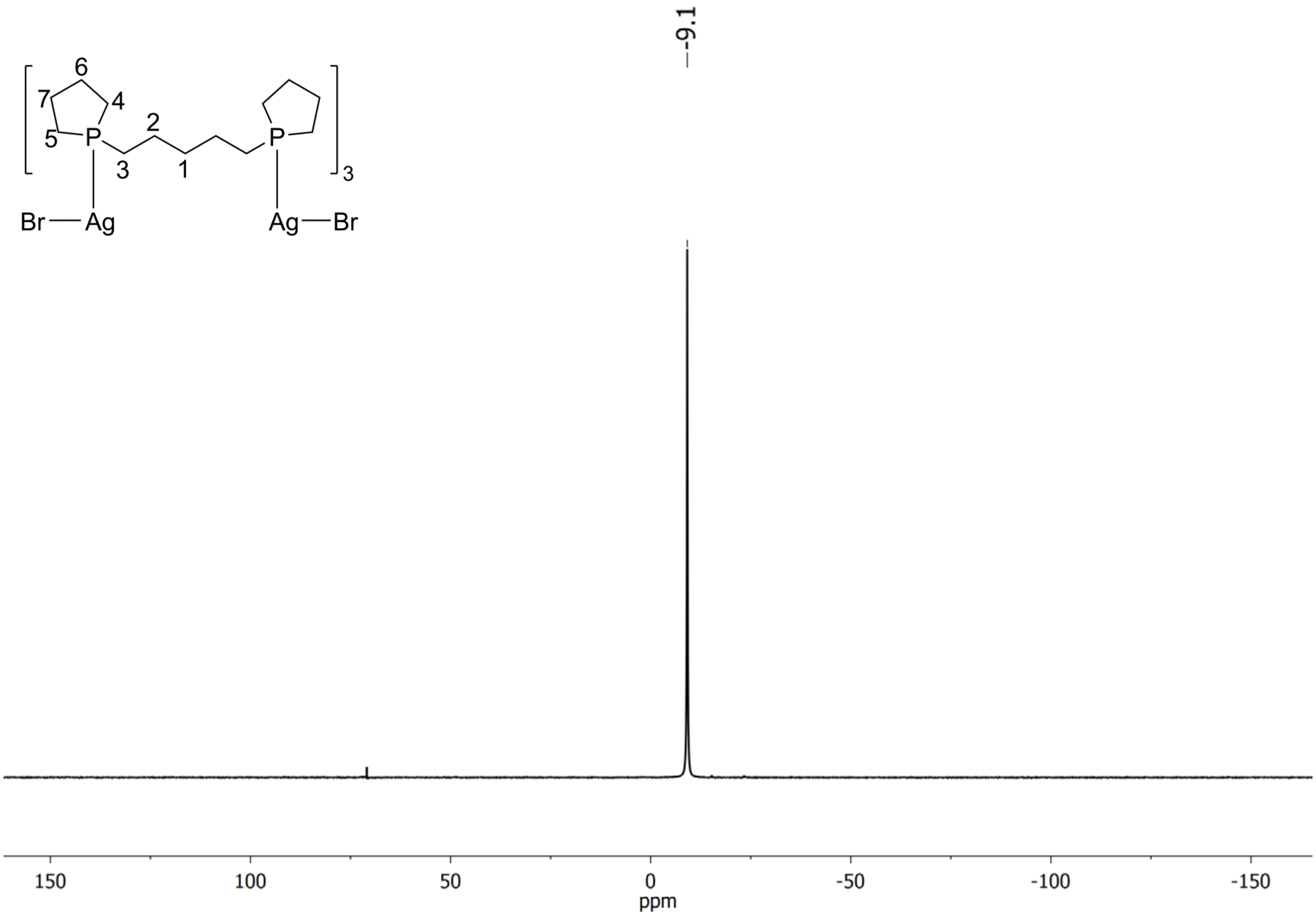

Fig S7-3. $^{31}P\{^{1}H\}$ NMR spectrum of complex **5** in $CDCl_3$

Generic Display Report

Analysis Info

Analysis Name	D:\Data\Service_Q2_16\Boar_C5_AgBr_3_2_1.d	Acquisition Date	5/2/2016 9:17:15 AM
Method	Service_ESI.m	Operator	oe
Sample Name	C5+AgBr (3:2)	Instrument	impact II
Comment	in DCM/MeOH		

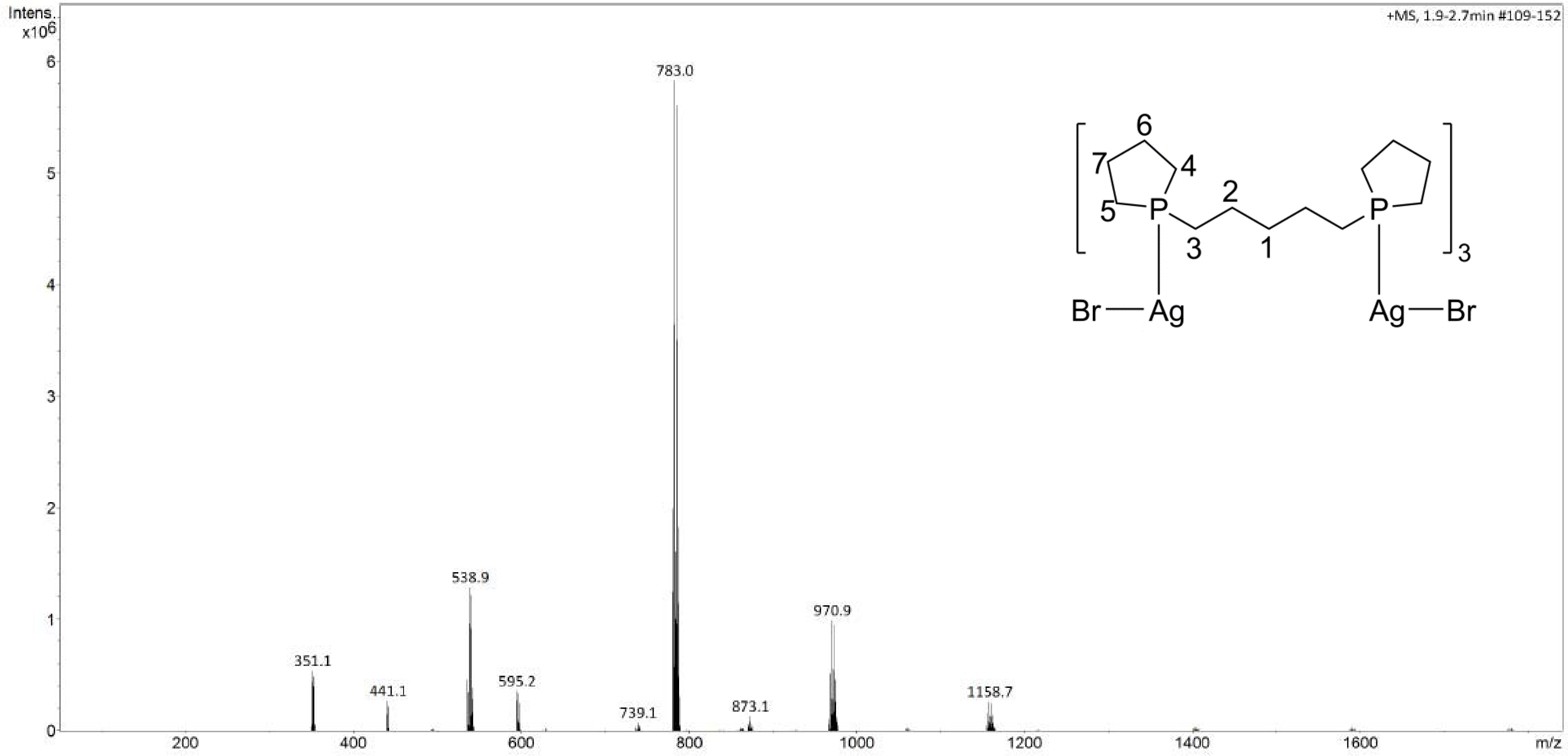

Bruker Compass DataAnalysis 4.2 printed: 5/2/2016 9:51:34 AM Page 1 of 1

Fig S7-4. MS (ESI(+), DCM/MeOH) of complex **5**

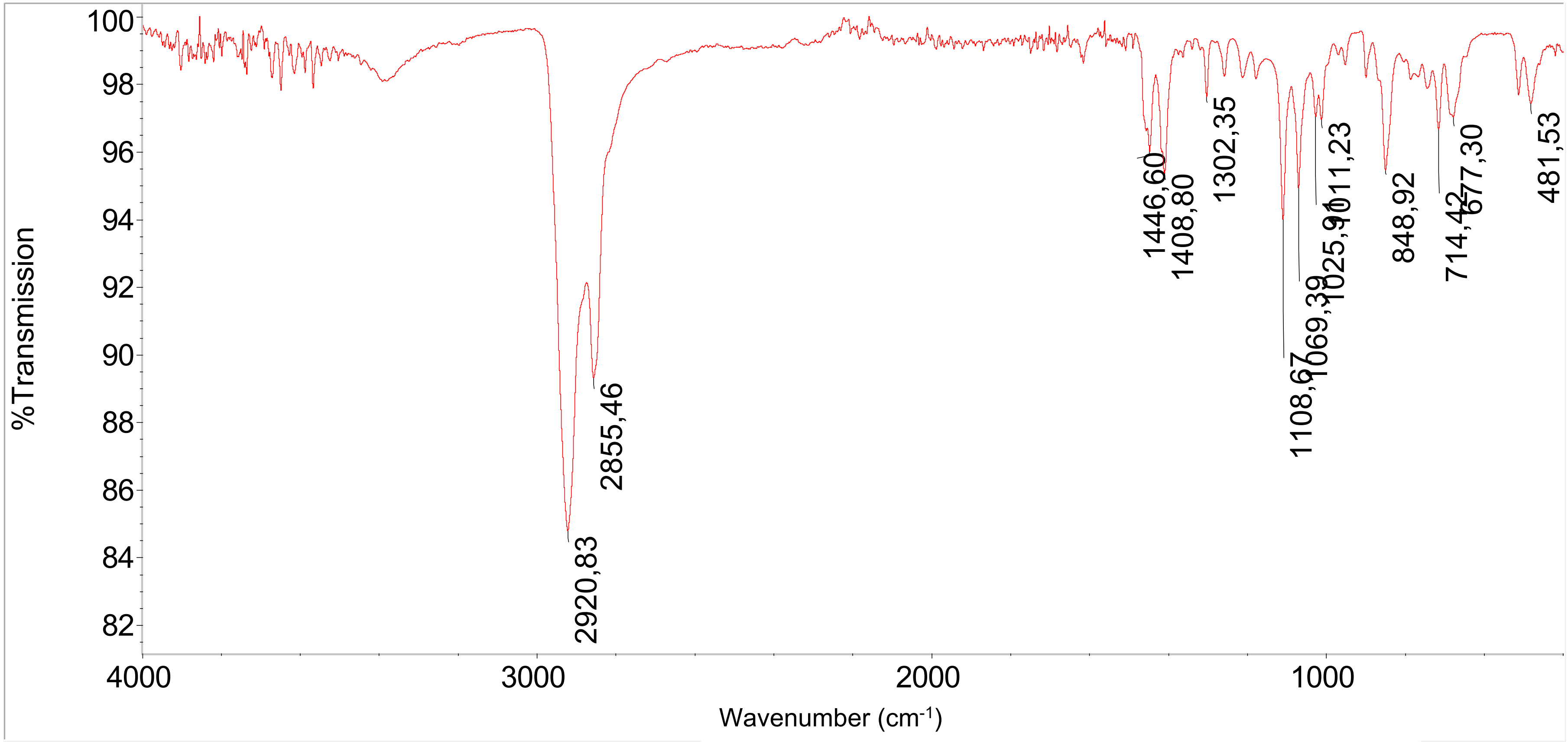

Fig S7-5. IR spectrum of complex **5**

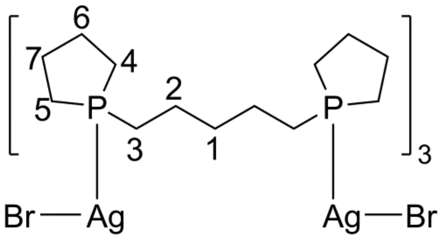

Supplement: Supplementary file 1 — Supporting Information [file ZAAC-646-915-s001.pdf]
